# NRSF/REST lies at the intersection between epigenetic regulation, miRNA-mediated gene control and neurodevelopmental pathways associated with Intellectual disability (ID) and Schizophrenia

**DOI:** 10.1038/s41398-022-02199-z

**Published:** 2022-10-10

**Authors:** Mouhamed Alsaqati, Brittany A. Davis, Jamie Wood, Megan M. Jones, Lora Jones, Aishah Westwood, Olena Petter, Anthony R. Isles, David Linden, Marianne Van den Bree, Michael Owen, Jeremy Hall, Adrian J. Harwood

**Affiliations:** 1 Neuroscience and Mental Health Research Institute, Hadyn Ellis Building, Cathays, Cardiff, CF24 4HQ UK; 2 MRC Centre for Neuropsychiatric Genetics and Genomics, Division of Psychological Medicine and Clinical Neurosciences (DPMCN), School of Medicine, Cardiff University, Cardiff, UK; 3 Lieber Institute for Brain Development, Johns Hopkins Medical Campus & Department of Psychiatry and Behavioral Sciences, Johns Hopkins University School of Medicine, Baltimore, MD USA; 4 School of Bioscience, The Sir Martin Evans Building, Museum Ave, Cardiff, CF10 3AX UK; 5 School of Mental Health and Neuroscience, Faculty of Health, Medicine and Life Sciences, Maastricht University, Maastricht, Netherlands; 6 Present Address: School of Pharmacy, KGVI Building, Newcastle University, Newcastle Upon Tyne, NE1 4LF UK

**Keywords:** Epigenetics in the nervous system, Stem cells, Psychiatric disorders

## Abstract

Genetic evidence indicates disrupted epigenetic regulation as a major risk factor for psychiatric disorders, but the molecular mechanisms that drive this association remain to be determined. EHMT1 is an epigenetic repressor that is causal for Kleefstra Syndrome (KS), a genetic disorder linked with neurodevelopmental disorders and associated with schizophrenia. Here, we show that reduced EHMT1 activity decreases NRSF/REST protein leading to abnormal neuronal gene expression and progression of neurodevelopment in human iPSC. We further show that EHMT1 regulates NRSF/REST indirectly via repression of miRNA and leads to aberrant neuronal gene regulation and neurodevelopment timing. Expression of a NRSF/REST mRNA that lacks the miRNA-binding sites restores neuronal gene regulation to EHMT1 deficient cells. Significantly, the EHMT1-regulated miRNA gene set not only controls NRSF/REST but is enriched for association for Intellectual Disability (ID) and schizophrenia. This reveals a broad molecular interaction between H3K9 demethylation, NSRF/REST regulation and risk for ID and Schizophrenia.

## Introduction

Genetic evidence points to an association of chromatin remodellers, mediators of epigenetic regulation, as a substantial risk factor for many common psychiatric disorders [1, 2]. Genome-wide association studies (GWAS) have revealed multiple risk loci for neurodevelopmental disorders (NDD) that are associated with genes encoding epigenetic regulators. These often encompass more than one condition, including Intellectual Disability (ID), autism spectrum disorders (ASD) and schizophrenia. Accordingly, alleles affecting epigenetic regulatory mechanisms are associated with a range of psychiatric symptoms, including cognitive deficits, autistic traits, and psychosis. Epigenetic-related risk alleles are linked with biological pathways that converge on chromatin regulation via control of nucleosome positioning and histone methylation, leading to altered gene transcription [3, 4].

Studies on high risk, loss of function (LOF) gene variants associated with NDD reinforce this view. Disruption of the SNF-2 family chromatin re-modellers CHD7 and CHD8 are strongly associated with ID and along with CHD2 confer risk for ASD [5]. Histone lysine methyltransferases (KMT) are key epigenetic regulators, and many are associated with NDD and psychiatric disorders. LOF mutations of MLL3 (KMT2C), MLL5 (KMT2E), ASH1L (KMT2H), SUV420H1 (KMT5B) and histone lysine demethylases (KDM), KDM5B and KDM6B are all associated with ASD [6–8]. LOF variants of the H3K4 methyltransferase SETD1A are associated with schizophrenia, developmental delay (DD) and ID [9]. Other KMT are linked to severe neurodevelopmental disruption and ID associated genetic syndromes; KMT2A with Wiedemann-Steiner Syndrome, KMT2D with Kabuki Syndrome and KMT1D with Kleefstra syndrome (KS). The latter is subject of this study and here referred by its common name, Euchromatic Histone-Lysine N-Methyltransferase 1 or EHMT1. KS is associated with autistic features, psychosis, and schizophrenia [10]. A recent study indicates an association of *de novo* postzygotic EHMT1 mutation and an ASD and neurocognitive dysfunctions in adults [11]. Although the genetic case for epigenetic regulation is well established, there is little knowledge of the downstream molecular mechanisms that link their actions to the underlying pathophysiology of the NDD. To address this question, we have investigated the mechanism by which reduced EHMT1 activity leads to an altered neurodevelopmental programme in both isogenic cell models of Kleefstra syndrome and patient-derived iPSC.

Canonical Kleefstra syndrome (KS) arises from a sub-telomeric microdeletion at 9q34, resulting in a heterozygous deletion of approximately ∼700-kb [12]. This region contains at least five genes, including *ZMYND19*, *ARRDC1*, *C9ORF37, EHMT1*, and *CACNA1B* [12, 13], however, the core clinical phenotypes are driven by haploinsufficiency of *EHMT1* [12]. Consistent with an epigenetic origin of KS, a broader Kleefstra syndrome phenotypic spectrum (KSS) is also associated with other chromatin modifiers, including MLL3 (KMT2C) [14]. EHMT1 is the primary enzyme for dimethylation of histone H3 at Lys9 residues (H3K9me2) [15] and is generally associated with transcriptional gene silencing [16].

Genetic manipulation of EHMT1 has been studied in *Drosophil*a and in rodent models at molecular, cellular, and behavioural levels. *Drosophila ehmt1* mutants exhibit decreased dendrite branching of sensory neurons and impaired short and long-term memory that is reversed by restoring EHMT expression [17]. *EHMT1*^−/+^ mice demonstrate cranial abnormalities, hypotonia and delayed postnatal growth [18]. Functionally they show deficits in fear extinction and novel object recognition [18–21]. In rodent primary neuron cultures EHMT1 regulates the dynamics of multiple neural processes, including synaptic scaling and response to addiction and stress [20, 22] and knockdown of Ehmt1 or the other KSS associated genes alter synaptic gene regulation and neuronal excitability. [23, 24]. To date, studies on KS patient-derived iPSC have focussed on differentiated, mature neurons, and like mouse studies they show altered neuronal activity, synaptic signalling, and network properties [25].

Here we identify a molecular biological pathway that is disrupted during early differentiation of human cellular models of KS. We show that EHMT1 regulates the transcriptional repressor NRSF/REST, a neuron-specific gene regulator [26–28]. This occurs via up-regulation of microRNA (miRNA)-mediated suppression of NRSF/REST protein synthesis. Analysis of the EHMT1-regulated miRNA expression profile, including miR-153, miR-26a and miR-142, indicates a broader association between EHMT1 regulated miRNA, NRSF/REST and both ID and schizophrenia. These gene regulatory abnormalities have substantial effects on neuronal gene expression and in vitro neurodevelopment, causing premature neurodifferentiation and neuronal dysfunction.

## Materials and methods

### Human iPSCs culture and neuronal differentiation

The IBJ4 human iPSC line derived from the BJ fibroblast cell line (ATCC; CRL-2522) was used, unless indicated. HiPSC were grown on matrigel (Corning) in Essential 8™ medium (ThermoFisher scientific) at 37 °C, 5% CO_2_ [29]. Medium was changed every day and cells were passaged using gentle cell dissociation reagent (Stemcell Technologies) or singularised using accutase (Stem Cell Technologies). hiPSCs differentiation to glutamatergic neurons was based on a modified method of Chambers et al. [30] using N2B27 (2/3 DMEM/F12; 1/3; neurobasal; B27-RA; N2; 1xPSG; 0.1 mM β-mecaptoethanol) + 100 nM SB431542 and 100 nM LDN193189, followed by a N2B27 with B27 + retinoic acid (with 10 µM DAPT for first 7 days) on PDL (Sigma)/laminin (Roch) at 200000 cells/cm^2^.

### Kleefstra syndrome (KS) hiPSCs generation

#### Generation of KS-patient hiPSCs

Two KS patients were selected: (i) a 22-year-old female patient, non-verbal (IQ not measurable), with a history of epilepsy (treated with carbamazepine and levetiracetam), hypotonia, anxiety disorder and depression; (ii) 20-year-old female patient, (FSIQ 53) diagnosed with ASD, specific phobia, psychotic symptoms, hypotonia, unprovoked seizures, and cardiac, mitral valve insufficiency. The participants were recruited as part of a research cohort on neurodevelopmental copy number variants at Cardiff University (the Defining Endophenotypes from Integrated Neuroscience [DEFINE] Study). Procedures included clinical and cognitive testing, where possible, and blood sampling for generation of iPSCs and were approved by the South-East Wales Research Ethics Committee. Where participants did not have capacity to consent, as in this case, a representative (next of kin) provided written informed consent on their behalf. Peripheral blood mononuclear cells (PBMCs) from each donor were reprogrammed using a CytoTune-iPS 2.0 Sendai reprogramming kit (A16517, ThermoFisher scientific) [31]. Karyotype analysis showed 46, XX normal diploid female karyotype (ISCN classification) and possessed a 9q34 deletion (Fig. S1B).

#### Mouse ES cells culture and neuronal differentiation

Analysis used two independent clonal populations of mESC *Ehmt1*^+/−^ cell lines mutant mouse ES cells (mESCs), obtained from the European Mouse Mutant Cell Repository Centre (EuMMCR); each had a single copy of a ‘knockout first’ conditional allele Ehmt1^tm1a(EUcOMM)Hmgu^ allele [32]. Control *(Ehmt*^flp^) a cell lines were generated using the flp-allele to restore a wild type gene. mESCs were grown on gelatin-coated plates in knockout DMEM (Gibco), supplemented with ESC certified FBS (Invitrogen), L-Glutamine (Gibco), 2-mercaptoethanol (Sigma) and ESGRO leukaemia inhibitory factor (LIF) (Chemicon) at 37 °C. Feeder-free neuronal differentiation was initiated in media lacking LIF for 4 days and 5 μM Retinoic acid was added to the culture until day 8. At this stage, cells were dissociated with 0.05% trypsin (Sigma) and seeded at 1.5 ×10^5^ per cm^2^ density on Poly‐D-ornithine (Sigma)/laminin (Roch) in N2 medium (Sigma) [33].

#### Molecular genetic manipulation of hiPSC lines

CRISPR-mediated mutagenesis of the *ehmt1* gene used a modified IBJ4 cell line, possessing the plasmid pAAVS1-PDi-CRISPRn (Addgene) inserted into the AAVS1 safe-harbour locus. This contains a Tet-inducible Cas9-nuclease, which was induced by 2 μg/ml Doxycycline (Dox) 24 h day before transfection with 10 pmol each of two ehmt1-specific synthetic gRNA (SygRNA), crRNA and tracrRNA using Lipofectamine CRISPRMAX (Life Technologies). Successful editing was confirmed by PCR amplification using the flanking primers 5‘-AGCAGCATCTCTCACCGTTT-3‘ and 5‘-CTTTTTCAGGTGGACGACTGG-3, size-separated by electrophoresis on a 4% agarose gel. The open CRISPR design tool (Sigma) was used to predict four potential off-target sites, which shared 3 base mismatches in the guide RNA (no 1 or 2 base mismatches were identified). PCR analysis demonstrated to be unmutated in our hiPSC lines (product sizes are shown in Table S2). A Tet-inducible REST gene was created by replacing the Cas9 gene of pAAVS1-PDi-CRISPRn with a REST cDNA sequence missing the 3’URT (GenBank BC132859.1). A synthetic NRSF/REST fragment (IDT Inc.) was subcloned into the linearised plasmid backbone vector in an isothermal Gibson assembly reaction (Gibson Assembly® Cloning Kit, New England BioLabs); Fig. S5C) [34]. Successful assembly was verified by sequencing, and by PCR where the custom construct was digested with AfIII and AgeI to release REST cDNA insert of ~3300 bp (Fig. S5D).

### Expression Analysis

qRT-PCR: Cells were lysed with QIAzol Lysis Reagent (Qiagen) and total RNA was extracted using the miRNeasy mini kit (reference 217004, Qiagen, Germany). For each sample, 1 µg of total RNA was reverse transcribed using the miScript II RT Kit (Qiagen). qRT-PCR analysis of miRNA used miScript SYBR Green PCR kit (218073; Qiagen, Germany) and mRNA analysis use QuantiTect SYBR Green PCR kit (Qiagen, Germany), All qRT-PCR reactions were performed in triplicate on a StepOnePlus™ Real-Time PCR System (Applied Biosystems) and relative expression calculated using the 2^−ΔΔCT^ method [35] with data -normalized to GAPDH and Colrf43 (see Table S3 for primer sequences).

Western analysis: Cells were washed with ice-cold PBS and lysed in ice-cold RIPA buffer (Sigma) and protease inhibitor cocktail (Sigma) or 30 min at 4 °C. Cell supernatants were collected by centrifuged at 21000 rcf at 4 °C, LDS sample buffer (NuPAGE) and sample reducing agent (NuPAGE) added and samples were heated at 95 °C for 5 min. 15 µg of protein per sample was separated by electrophoresis on 4–12% Bis‐Tris Plus Gels (Life Technologies), transferred to nitrocellulose, blocked solution 5% (w/v) powdered milk in Tris-buffered saline containing 0.1% (v/v) Tween 20 (TBST) for 60 min at RT and incubated overnight at 4 °C with primary antibody against REST (1:500) (ab75785, ab21635, Abcam), H3k9me2 (1:500) (ab1220, Abcam), MAP2 (1:750) (MAB8304, R&D Systems, Minneapolis, MN) or Caspase-3 (1:500) (9662, Cell Signaling Technology, USA) diluted in the blocking solution. After washing in TBST, blots were incubated with an appropriate IRDye®-conjugated secondary antibody (LI-COR) and visualised/quantified with a Licor/Odyssey infrared imaging system (Biosciences, Biotechnology). All data normalization was GAPDH.

### miRNA-seq and analysis

The method used to amplify RNA was adapted from Abruzzi et al. [36] and was performed using TruSeq® Small RNA Library Prep kit (Illumina, USA). Total RNA was extracted from the IBJ4 line with or without UNC0638 treatment (untreated control is treated with the same volume of DMSO as treated sample) and ligated to 3’ polyadenylated and 5′-adaptors, followed by reverse transcription, PCR amplification. cDNA and size-selection on 3% certified™ low range ultra-agarose (Bio-Rad Laboratories Ltd) in TBE buffer. Quality of purified miRNA libraries (QIAquick Gel Extraction Kit; Qiagen, Germany) were confirmed by Bioanalyzer (Agilent Technologies) and by Qubit (Thermo Fisher Scientific) (Fig. S4B) and sequenced on an Illumina HiSeq 4000 using single-end 50 base pair reads to deliver a minimum of 35 million mapped reads per sample (Fig. S4). Single-end sequence reads were trimmed with Trimmomatic [37], assessed for quality using FastQC and mapped to the human GRCh38 reference genome using STAR [38]. Counts were assigned to mirbase miRNAs using featureCounts [39] and the GRCm38.84 Ensembl gene build GTF. Differential gene expression (DEG) analyses used the DESeq2 package [40] and miRNA expression was FPKM-normalised, discarding miRNAs lacking significant differences between control and UNC0638-treatment at ≥2.5 fold change cut-off threshold (significance: adj.*p*val <0.05, Benjamini-Hochberg correction for multiple testing). miRNA-seq data have been deposited to ArrayExpress under accession number E-MTAB-10480.

A crossover analysis between miRNA gene targets and disease-associated genes was performed for Intellectual Disability, Schizophrenia and Autism Spectrum Disorder (ASD). Genes associated with Intellectual Disability and ASD (HP:0001249 and HP:0000717 respectively) were shortlisted from the DECIPHER database (DDG2P - V11.2), whilst Schizophrenia associated genes were shortlisted from the GWAS Catalog - EMBL-EBI (V1.0.2). Predicted miRNA target genes were determined using the miRDB database (V6.0). To account for miRNA target score, gene crossover probability was calculated for each miRNA in ‘R’ (V4.0.4), using noncentral hypergeometric distribution, with *P* < 0.01 considered statistically significant. Crossover probability between disease-associated miRNAs and REST targeting miRNAs was assessed for each disorder by calculating hypergeometric probability, with *P* < 0.05 considered statistically significant.

### Chromatin immunoprecipitation (ChIP)-qRT-PCR

Chromatin immunoprecipitation was performed as previously described [41]. Briefly, hiPSC suspensions c in serum-free media were crosslinked in 1% Formaldehyde (Sigma) for 10 min at RT and quenched by 0.125 M Glycine (Sigma) for 5 min at RT. PBS-washed cells were resuspended in cell lysis buffer (10 mM Tris-HCl pH 8.0, 10 mM NaCl, 0.2% Igepal CA-630, 10 mM sodium butyrate, 50 μg/mL PMSF, 1 μg/mL leupeptin) for 10 min on ice, nucleic collected by centrifugation for 5 min at 4 °C, resuspended in nuclear lysis buffer (50 mM Tris-HCl pH 8.1, 10 mM EDTA, 1% SDS, 10 mM sodium butyrate, 50 μg/mL PMSF, 1 μg/mL leupeptin) and diluted with immunoprecipitation dilution buffer. Chromatin was sheared to a fragment size of ∼200–600 bp by sonication cycles of 30 s ON/30 s OFF at HIGH power by the Bioruptor® PLUS (Fig. S4A). The chromatin was precleared by adding rabbit IgG (Merck Millipore)p and protein G-agarose suspension (Roche), followed by ChIP with10µg of di-methylated histone H3 (ab1220, Abcam) [42] or immunoglobulin control were incubated with the chromatin overnight at 4 °C. and then protein G-agarose (Roche) added. Protein G-agarose conjugates were pelleted, washed twice with IP wash buffer 1 (20 mM Tris-HCl pH 8.1, 50 mM NaCl, 2 mM EDTA, 1% Triton X-100, 0.01% SDS), once with 750 μL of IP wash buffer 2 (10 mM Tris-HCl pH 8.1, 250 mM LiCl, 1 mM EDTA, 1% IGEPAL CA630, 1% deoxycholic acid) and twice with 10 mM Tris-HCl 1 mM EDTA pH 8.0 and eluted in IP elution buffer (100 mM NaHCO3, 0.1% SDS). Samples were treated with RNase A (MilliporeSigma) and 5 M NaCl at 65°°C for 6 h, then proteinase K (ThermoFisher scientific) at 45 °C overnight. The DNA in both ChIP and input samples was purified using QIAquick PCR Purification Kit (28104, Qiagen, Germany), and subjected to qRT-PCR with primer sets are listed in Table S4. Amplified material was detected using QuantiTect SYBR Green PCR kit (Qiagen, Germany) on a StepOnePlus™ Real-Time qPCR System (Applied Biosystems) and comparison of ChIP product compared to input sample, for NRSF/REST and miR.

### Cell analysis

Immunocytochemistry. Cells were washed with PBS; fixed in 3.7% PFA for 20 min at 4 °C, blocked for 1 h in PBS with 0.3% Triton-X-100 (PBS-T) and 5% donkey serum for 1 h, before incubation with primary antibodies in PBS-T with 5% donkey serum overnight at 4 °C. Secondary antibodies were applied in PBS-T for 1.5 h at RT, counterstained with DAPI (Molecular Probes) and mounted in DAKO fluorescent mountant (Life Technologies). Samples were imaged on a Leica DMI6000b fluorescent microscope or analysed using a CX7 High-Content Screening (HCS) Platform (Thermo Fisher Scientific). Primary antibodies were as follows: Nanog (1∶200, 4903, Cell Signaling Technology, USA), Oct-4 (1∶200, 2750, Cell Signaling Technology, USA), Sox2 (1:200, 3579, Cell Signaling Technology, USA), NeuN (1:250, MAB377, Sigma). Secondary antibodies used were: Alexa 594-conjugated donkey anti-rabbit (1∶1000, Invitrogen, A21207), Alexa 488-conjugated donkey anti-rabbit (1∶1000, Invitrogen, A21206) and Alexa 488-conjugated donkey anti-mouse (1∶1000, Invitrogen, A21202). Cell counts and intensity measurements from at least 3 replicates were used for statistical analysis. For calcium imaging, neurons were grown in BrainPhys basal on coverslips at 50000 cells/cm^2^ for a week and analysed for spontaneous calcium events using Cal-520™ AM (Abcam) and 20% pluronic acid and videoed on a Zeiss Axio Observer inverted microscope (40× objective) using Zeiss Zen software. Region of interests (ROIs) were recorded for 5 min per experiment at a frame rate of 10 Hz and 1024 × 1024 pixel resolution. Image stacks were analysed by Fuji [43], NeuroCa [44], and FluroSNAAP [45] software packages. Results were imported into Prism 7.0 for statistical evaluation.

### Statistical analysis

Prism 7.0 (GraphPad Software) was used for the statistical analysis. Data shown are the mean ± SEM. with *P* < 0.05 considered statistically significant. Two-tailed unpaired t-tests were used for comparisons between two groups. Group differences were analysed with one-way analysis of variance (ANOVA) followed by Tukey’s multiple comparisons test. Data distribution was assumed to be normal, but this was not formally tested.

## Results

### Loss of Ehmt1 reduces expression of NRSF/REST and increases expression of REST-target genes

**Fig. 1:**
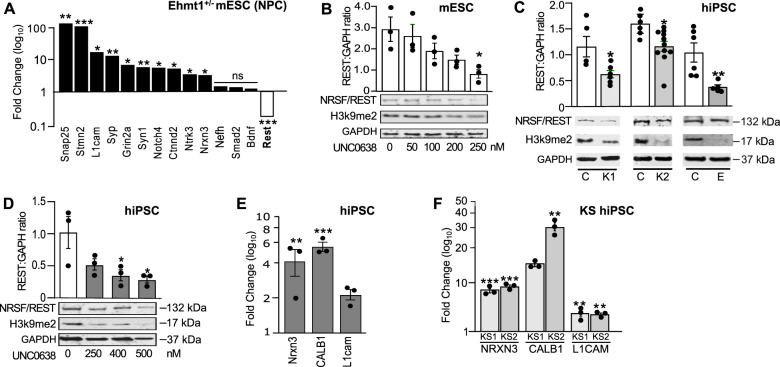
Expression of NRSF/REST and its target-genes in reduced EHMT1 activity and KS patient iPSC. **A** Gene analysis using qRT-PCR of Ehmt1^−/+^ mESC-derived NPCs compared to wild type cells. Expression of NRSF/REST mRNA was decreased while neuronal genes regulated by REST were elevated in Ehmt1^−/+^ cells. Mean fold change over wild type NPCs, n ≥ 3 independent experiments, log_10_ scale axis. **B**–**D** Western blot analysis of NRSF/REST and H3K9me2 protein in pluripotent cells: (**B**) mESC following treatment with range of UNC0638 concentrations for 48 h; (**C**) KS patient (2 patients; KS1 and KS2) and EHMT1^−/+^ iPSC (denoted as **E**) compared to the isogenic control hiPSC; (**D**) Nonpatient control iPSC treatment with range of UNC0638 concentrations for 72 h. Plotted as Western band intensity normalized to GAPDH, *n* ≥ 3. **E**, **F** Expression of REST-target genes NRXN3, Calbindin (CALB1) and L1Cam in pluripotent hiPSC, measured by qRT-PCR: (**E**) control hiPSC treated with 250 nM UNC0636 for 72 h (**E**), and (**F**) pluripotent KS patient iPSC. Mean Fold change greater than untreated control hiPSC (log_10_ axis), *n* ≥ 3 independent experiments. Data were presented as Mean±SEM and analysed by student’s t-test or One-way ANOVA with post hoc comparisons using Dunnett’s multiple comparisons test comparing to control samples. *P < 0.05, **P < 0.01, ***P < 0.001.

We initially examined the effect of *Ehmt1* hemizygosity on neurodevelopment-specific gene expression in mouse embryonic stem cells (mESC). mESC *Ehmt1*^+/−^ and *Ehmt*^flp^ control cell lines were cultured to neural progenitor cell stage (NPC) and expression profiles of 65 genes examined using qRT-PCR. A notable feature of the resulting data was a greater than sixfold decrease of *Rest* mRNA, one of only 3 genes showing a decrease in mESC *Ehmt1*^+/−^ compared to control lines (Fig. 1A; Table S1). This was accompanied by significant increased expression of 10 out of 13 Nrsf/Rest-repressed genes present in the study. Based on these data we proposed that Ehmt1 may regulate neuronal gene transcription via control of Nrsf/Rest. To pursue the hypothesis, we treated wild type mESC with UNC0638, a selective inhibitor of EHMT histone methyltransferases [46]. As Nrsf/Rest is expressed in pluripotent stem cells, we tested mESC in the pluripotent state by treatment for 48 h with UNC0638. Western blot analysis showed a dose-dependent decrease of H3K9me2, with a half-maximal change at 200 nM (Fig. 1B). This was accompanied by a similar dose-dependent decrease in NRSF/REST protein.

To establish whether this observation is common to KS patients, we generated hiPSCs from two patients (Fig. S1). Both patient lines showed an approx. Twofold decrease in H3K9m2, accompanied by an equivalent decrease of NRSF/REST protein (Fig. 1C). This is likely to be solely due to loss of EHMT1 activity, as EHMT2 protein expression is unaltered (Fig. S1C). As the microdeletion in KS patients ablates multiple genes, we employed CRISPR-Cas9 to introduce a 56-bp deletion in exon 12 of *EHMT1* gene to create a hemizygous *EHMT1* knockout hiPSC line (*EHMT1*^−/+^), which would be isogenic with the parental wild type control cells. This deletion caused a 50% loss of EHMT1 protein (Fig. S2) and an accompanying reduction of H3K9me2 expression (Fig. 1C). As for KS patient iPSC and mESC, reduced EHMT1 activity again led to a significant reduction in the expression of NRSF/REST protein in *EHMT1*^−/+^ cells. Finally, UNC0638 treatment of wild-type hiPSCs caused a dose-dependent reduction of NRSF/REST protein, with half-maximal change at 250 nM (Fig. 1D).

To confirm that changes to NRSF/REST protein lead to altered gene transcription, we examined expression in undifferentiated, pluripotent cells of the NRSF/REST target genes NRXN3, Calbindin and L1CAM [47–49], which initiate their expression during early stages of neurodevelopment, but are not reported to be present in pluripotent cells. Increased gene expression was induced in non-patient hiPSC following 72 h of 250 nM UNC0638 treatment (Fig. 1E). Furthermore, hiPSC lines from both KS-patients showed a 5-fold or greater expression of these genes than control cultures (Fig. 1F). Collectively, these mouse and human data indicate that EHMT1 regulates the level of NRSF/REST of pluripotent stem cells, and when reduced directly elevates the expression of its downstream target genes.

### EHMT1 regulates NRSF/REST via suppression of miRNA-associated that are associated with psychiatric disorders

**Fig. 2:**
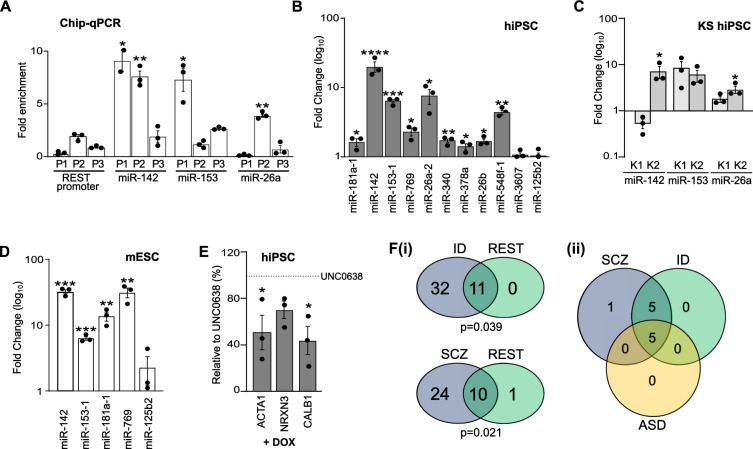
EHMT1 suppresses expression of miRNA. **A** ChIP-qRT-PCR analysis of H3K9me2 modification within 3 regions of NRSF/REST promoter (P1, P2, P3) and surrounding (P1, P2) and distal to (P3) the Translational Start Sites (TSSs) of miR-142, miR-153 and miR-26a promoters of pluripotent hiPSC (see Fig. S3). ChIP was performed with an anti-H3K9me2 antibody, and H3K9me2 enrichments were analysed by qRT-PCR. Enrichment is plotted as increase relative to the input DNA in specific genomic regions in the absence of ChIP antibody primer sets are listed in Table S4. **B**–**D** Validation of miRNA-seq data by qRT-PCR to confirm miRNA-seq results: (**B**) UNC0638-treated hiPSC; (**C**) KS-patient iPSC; (**D**) UNC0638-treated mECS. The qRT-PCR data were shown as Mean±SEM and analysed by One-way ANOVA with post hoc comparisons using Dunnett’s multiple comparisons test comparing to control samples. *P < 0.05, **P < 0.01, ***P < 0.001, ****P < 0.0001, *n* ≥ 3. **E** Relative mRNA abundance measured by qRT-PCR of the REST-target genes ACTA1, NRXN3, and Calbindin1 (CALB1) in pluripotent hiPSCs treated with UNC0638. Induction of RESTΔUTR with doxycycline (DOX) suppresses the UNC0638-induced gene expression. Results are plotted as % of mRNA abundance in the absence of DOX, shown as dotted line. **F**, **i** Venn diagrams showing association between miRNAs following UNC0638 treatment and those associated with Intellectual Disability (ID) and schizophrenia (SCZ). Crossover probability was assessed for each disorder by calculating hypergeometric probability, with *P* < 0.05 considered statistically significant; (**ii**) Venn diagram to show overlap of REST targeting miRNAs between ID, SCZ and ASD.

Conventionally, H3K9me2 is considered to be a transcriptional repressor [50], yet we observed that reduced EHMT1 activity leads to decreased NRSF/REST protein. We found no evidence for H3K9me2 at the *NRSF/REST* promoter using ChIP-qRT-PCR (Figs. 2A and S3), arguing against direct regulation by EHMT1 of *NRSF/REST* gene transcription. We therefore considered the potential of a de-repression mechanism acting via suppression of an intermediate NRSF/REST repressor. MicroRNAs (miRNAs) are ∼22-nt noncoding RNAs expressed in a wide range of eukaryotic organisms and play a critical role in the regulation of gene expression at the post-transcriptional level. They have crucial roles at key stages in the development of the nervous system [51] and several brain-related miRNAs, including miR-142, miR-153 and miR-9 have been shown to target NRSF/REST mRNA [49]. This offers a mechanism to connect repressive EHMT1 histone methylation at the genome level to control of NRSF/REST protein and its subsequent regulation of neuronal gene expression.

We conducted an unbiased search for miRNAs regulated by EHMT1 by miRNA-seq of wild-type hiPSC in the presence or absence of UNC0638. We detected 56 miRNAs with greater than a 2.5-fold increase of expression when EHMT1 is inhibited (Fig. S4). 11 of these miRNAs were predicted to target NRSF/REST mRNA based on miRDB database, and 9 replicated by qRT-PCR analysis of UNC0638-treated hiPSC, including miR-142, miR-153-1, miR-26a-2, miR-548f-1 (upregulated by 20.6 ± 3.50, 6.70 ± 0.19, 7.95 ± 1.908 and 4.62 ± 0.35-fold, respectively; Fig. 2B). To validate this further we examined miR-142, miR-153-1, miR-26a-2 in our KS-patient iPSC, and found up-regulation of all three miRNA, with the exception of miR-142 in KS patient 1 (Fig. 2C). Furthermore, the association between EHMT1 activity and miRNA expression was conserved in mESC as expression of miR-142, miR-153-1, miR-181a and miR-769 were all elevated after UNC0638 treatment (Fig. 2D). To investigate direct linkage between these NRSF/REST-regulating miRNAs and EHMT1 activity we examined their association with H3K9me2-modfied chromatin, we further re-probed our H3K9me2-ChIP to show that in contrast to the NRSF/REST gene itself, H3K9 dimethylation was present at TSS of miR-142, miR-153 and miR-26a miRNA genes (Fig. 2A). Our combined gene expression and ChIP analysis supports an indirect regulation of EHMT1 on NRSF/REST via suppression of miRNA transcription.

To test our mechanistic hypothesis, we introduced a doxycycline-inducible (“Tet-on”) expression plasmid into the AAVS1 safe harbour site of wild-type hiPSC that expresses a recombinant NRSF/REST cDNA (RESTΔUTR) that lacks the miRNA-target region within its 3’UTR (Fig. S5). The rationale for this strategy is that removing all miRNA target sites in the NRSF/REST mRNA would overcome the redundancy due to the presence of multiple EHMT1-regulated miRNAs. For pluripotent cells in the presence of UNC0638, but absence of doxycycline, we observed an elevation of ACTA1, NRXN3 and Calbindin gene expression, which was blocked by doxycycline-induced expression of RESTΔUTR (Fig. 2E). This result demonstrates that uncoupling of NRSF/REST expression from its miRNA regulators is sufficient to overcome the effects of reduced EHMT1 activity.

Given the potential association between EHMT1 activity and patient diagnosis that extends across a range of NDD, we examined the relationship between the up-regulated miRNA gene set due to reduced EHMT1 activity and GWAS data for ID, schizophrenia and ASD. Remarkably, 43 and 34 of the 56 up-regulated miRNA have genetic association with ID and schizophrenia respectively (Table S5). In contrast, only 15 ASD-associated miRNA were up-regulated, all of which overlapped with the ID gene set and 13 of which were also associated with schizophrenia (Fig. S6). Of the up-regulated miRNA gene sets that are known NRSF/REST regulators, there was significant enrichment for those associated with ID (all 11 miRNA) and schizophrenia (10 of 11 miRNA), but not ASD (5 of 11 miRNA) (Figs. 2F and S6). Furthermore, the five NRSF/REST targeting miRNA associated with ASD were present in both ID and schizophrenia overlaps (Fig. 2F), giving a core miRNA gene set of miR-26a, miR-26b, miR-153, miR-181a and miR-548. This analysis suggests a broad association of a miRNA-mediated NRSF/REST regulatory pathway and psychiatric risk, particularly for a diagnosis of ID and schizophrenia.

### Reduced EHMT1 activity accelerates neuronal differentiation

**Fig. 3:**
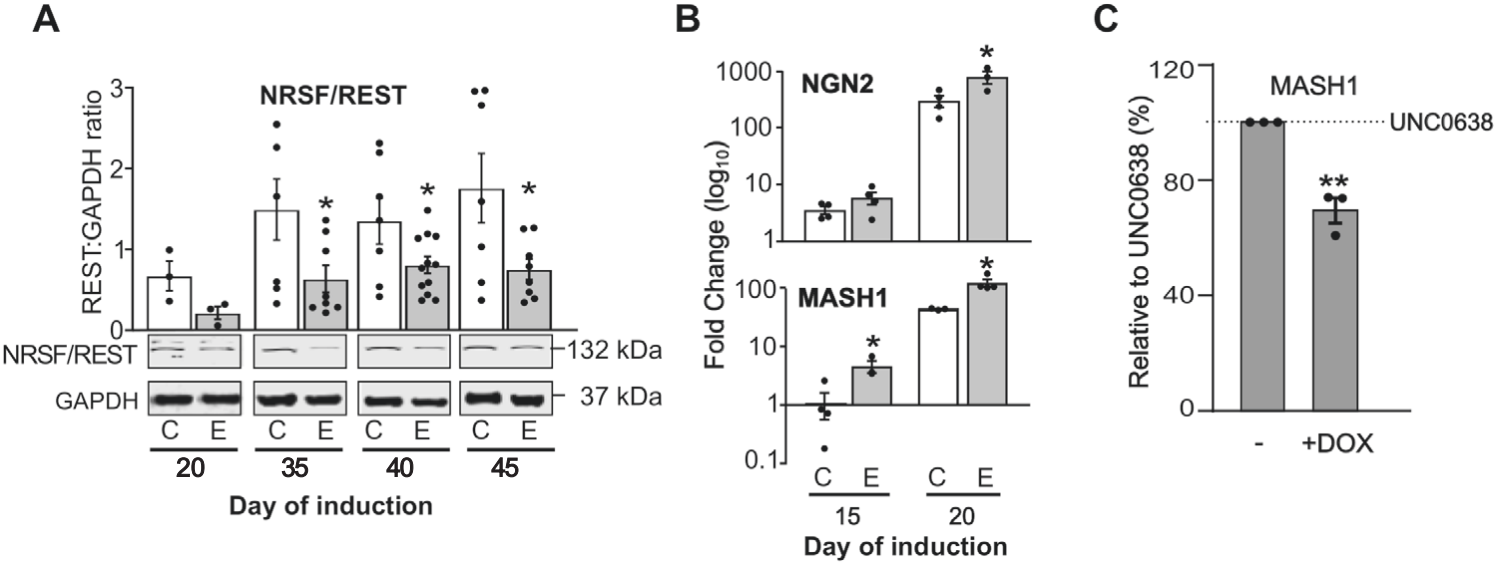
Reduced EHMT1 activity elevates REST-target gene expression during neuronal development. **A** Western blot analysis of NRSF/REST protein expression in hiPSCs-derived neurons at Day 20 (NPC stage) and maturing neurons at Days 35, 40 and 45 of differentiation. NRSF/REST protein was decreased in EHMT1^−/+^-derived neurons. Quantification of Western blot analysis was performed by normalization to GAPDH. A representative image from at least three independent experiments is shown, with all vales shown on the graph above. **B** Time-course qRT-PCR analysis at Days 15 and 20 of differentiation to examine changes in the expression of lineage-specific REST-target genes NGN2 and MASH1 in EHMT1^−/+^-derived neurons compared to their isogenic control hiPSCs. Relative changes is expressed as mean fold change over pluripotent cells, *n* ≥ 3 independent experiments. **C** Relative mRNA abundance measured by qRT-PCR of MASH1 in wild type hiPSCs treated with UNC0638. Induction of RESTΔUTR with doxycycline (DOX) suppresses the UNC0638-induced MASH1gene expression. Data were presented as Mean±SEM and analysed by student’s t-test or One-way ANOVA with post hoc comparisons using Dunnett’s multiple comparisons test comparing to control samples.

We investigated whether the relationship between reduced EHMT1 activity and NRSF/REST protein is maintained beyond the pluripotent cell state and persists into neurodevelopment. Mutant *EHMT1*^+/−^ hiPSCs and their isogenic wild-type controls were differentiated into neurons using a standard dual-SMAD inhibition protocol [30] and NRSF/REST protein levels sampled at time points that span neuronal differentiation (Fig. 3A). NRSF/REST protein was significantly lower during differentiation of *EHMT1*^−/+^ mutant cells compared to isogenic controls. This indicates that the effect of EHMT1 on NRSF/REST protein persists from early NPC stage into neuronal differentiation. The NRSF/REST reduction in differentiating *EHMT1*^−/+^ iPSC was accompanied by increased expression of the human orthologues of the target genes MASH1 and NGN2, which are first expressed in NPC [48, 52] (Fig. 3B). Expression of MASH1 in control hiPSCs derived NPCs was also increased in the presence of UNC0638 and suppressed by doxycycline-mediated induction of RESTΔUTR mRNA (Fig. 3C).

**Fig. 4:**
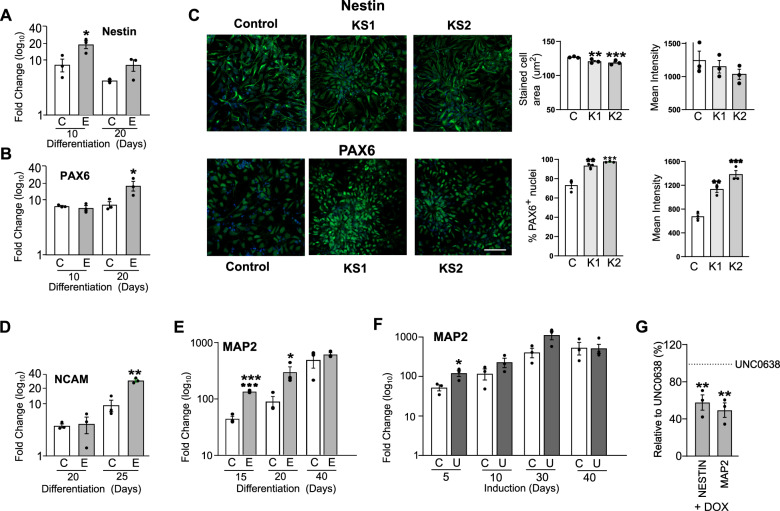
Reduced EHMT1 activity results in accelerated neuronal development. qRT-PCR analysis at days 10 and 20 of differentiation to examine the expression of the neural progenitor markers, (**A**) Nestin and (**B**) PAX6 in EHMT1^−/+^-derived NPC relative to expression in the isogenic control. **C** Cell staining of two KS patient iPSC developed to NPC (Day 20) stage and stained for Nestin and PAX6 protein. Scale Bar, 50 μm. Graphs show quantitation of protein expression as: stained cell area (Nestin); stained (PAX6^+^) and mean fluorescence intensity, with DAPI nuclear counterstain. **D** qRT-PCR analysis of NCAM at days 20 and 25 of early stage differentiated neurons in EHMT1^−/+^-derived relative to expression in the isogenic control. **E** qRT-PCR analysis at days 15, 20 and 40 of differentiation to examine changes in the expression of the neuronal marker MAP2 in EHMT1^−/+^-derived neurons relative to their expression in the isogenic control neurons. **F** UNC0638 (250 nM) induced expression of MAP2 in differentiating wild-type hiPSC-derived neurons, monitored by qRT-PCR analysis over a time course of 5, 10, 30 and 40 days of treatment. **G** Relative mRNA abundance measured by qRT-PCR of Nestin and MAP2 in wild-type hiPSC treated with UNC0638. Induction of RESTΔUTR with doxycycline (DOX) suppresses the UNC0638-induced MASH1 gene expression. *n* ≥ 3 independent experiments. Data were presented as Mean±SEM and analysed by One-way ANOVA with post hoc comparisons using Dunnett’s multiple comparisons test comparing to control samples.

These results suggest that EHMT1 acts via NRSF/REST as a negative regulator of neuronal gene expression to prevent premature neurodevelopment. To confirm that dysregulation of NRSF/REST-mediated gene repression subsequently impacts on neurodifferentiation in general, we investigated the non-NSRF/REST target genes, PAX6 and Nestin [53, 54]. These genes were upregulated at Day 10 and 20 of neuronal differentiation as EHMT1^+/−^ cells transit from the NPC stage (Fig. 4A, B). Likewise, cell staining of differentiated iPSC demonstrated an increase in PAX6 and Nestin in KS-patients (Fig. 4C). To probe neurodifferentiation further, we examined the expression of NCAM and MAP2 (microtubule-associated protein 2) proteins, which are expressed in all neurons [55]. Both genes were up-regulated during the early stages of neuronal differentiation in *EHMT1*^+/−^ cells during NPC stages, indicative of a rapid transition through the progenitor cell state and into full neuronal differentiation (Fig. 4D, E). As also seen with other genes, elevated MAP2 expression was induced in differentiated wild-type hiPSC by UNC0638 inhibition of EHMT1 (Fig. 4F). Finally, elevated expression of both Nestin and MAP2 was suppressed by doxycycline-induced RESTΔUTR expression demonstrating NRSF/REST dependency (Fig. 4G). These results are indicative of indicate accelerated neuronal differentiation with reduced EHMT1 activity, commencing as cells leave the pluripotent cell state and continuing during the formation of mature neurons.

### Reduced EHMT1 activity is associated with increased apoptosis and aberrant neuronal function

**Fig. 5:**
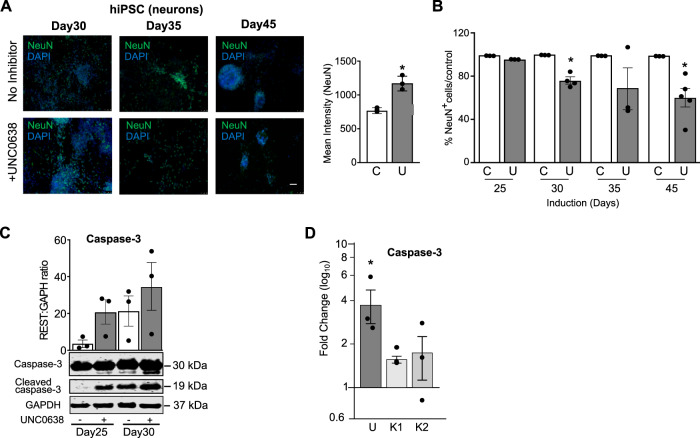
Adjustment of neuronal cell numbers during later differentiation. **A** Representative immunocytochemistry images of hiPSCs-derived neurons treated with UNC0638 (250 nM) at days 30, 35 and 45 of differentiation stained with NeuN (green) and counterstained with DAPI (blue). Scale Bar, 50 µM. Graph shows quantitation of NeuN expression (mean average intensity of cell nuclei, all data shown) at day 35 time point. **B** Total number of NeuN-cells at days 25, 30, 35 and 45 of differentiation in the absence and presence of UNC0638, n ≥ 3 independent experiments. **C** Western blot analysis at days 25 and 30 to examine the expression of cleaved-caspase-3 in the presence and absence of UNC0638 (250 nM). Top panel shows the expression of uncleaved caspase-3 (inactive), middle panel shows the expression of cleaved-caspase-3 (activated) and bottom panel shoes expression of GAPDH. Quantification of Western blot analysis was performed by normalization to GAPDH, *n* ≥ 3. **D** qRT-PCR analysis to examine the change in the expression of caspase-3 in hiPSCs-derived neurons treated with UNC0638 (250 nM). The expression of caspase-3 increased in UNC0638-treated neurons and patient iPSC compared to untreated neurons. Fold change over untreated neurons (mean). *n* ≥ 3 independent experiments. Data were presented as Mean±SEM and analysed by student’s t-test or One-way ANOVA with post hoc comparisons using Dunnett’s multiple comparisons test comparing to control samples.

Although we observed a consistent pattern of rapid neurodevelopment in cells with reduced EHMT1 activity in early developmental stages by Day 40 these differences appeared to be lost (Figs. 4E, F and S8). Previous reports described mice lacking NRSF/REST as having a transient increase in neurogenesis but eventually decreased of neuronal numbers [56]. We therefore investigated the impact of the prolonged inhibition of EHMT1 activity on neuronal differentiation, monitoring neuronal cell number using cell staining with the nuclear protein, NeuN, a marker of mature neurons. Wild-type cells were differentiated to neurons in the presence and absence of UNC0638 and sampled at Days 25, 30, 35 and 45 (Fig. 5A). At Day 35, we observed an elevation level of NeuN protein in stained cells Fig. 5A), consistent with our observations for NPC and early-stage neurons, however, cultures tracked over an extended induction period showed a progressive decrease in the proportion of NeuN-positive cells in the treated cells compared to untreated controls (Fig. 5B). By Day 45, UNC0638-treated cell cultures had 50% of the number of NeuN positive cells compared to untreated controls. This may explain the levelling of MAP2 expression observed between control and cells with reduced EHMT1 at later developmental time points, as the expression per cell may be higher, but the overall number of cells less (Fig. 4E, F).

During neurodevelopment, elevated rates of neurogenesis can may be balanced by decreased neuronal cell survival [57]. To determine the mechanism underlying the neuronal loss, we examined Caspase-3 activation in UNC0638-treated cells in relation to the control. At days 25 and 30 of neuronal differentiation, which are the time points preceding the reduction in cell number, we detected an elevated Caspase-3 cleavage to the activated form in UNC0638-treated cells (Fig. 5C). The increase in Caspase-3 activation was accompanied by increased Caspase-3 gene expression in control neurons treated with UNC0638 (Fig. 5D). These observations support the hypothesis that in EHMT1^−/+^ cells the reduction in the number of cells stained with NeuN and the neuronal gene expression may be due to an induction of programmed cell death.

**Fig. 6:**
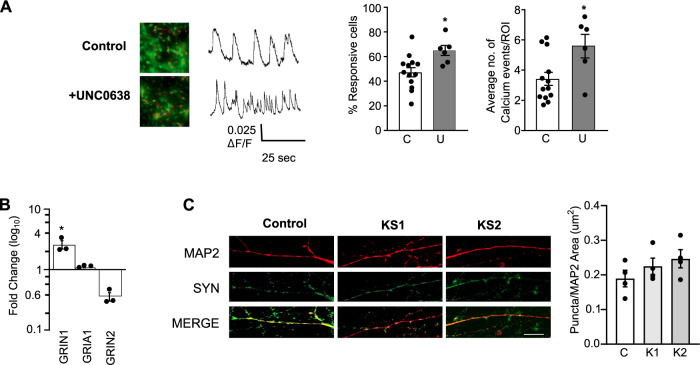
Reduced EHMT activity leads to aberrant neuronal activity. **A** Calcium imaging of hiPSCs-derived neurons. Differentiated neurons were analysed for spontaneous calcium events using Cal-520tm AM staining. Neurons were treated with UNC0638 for two weeks and calcium imaging was performed around day 50 of differentiation to compare the number of calcium event frequencies between control neurons and those treated with UNC0638. Representative images and traces of calcium influx of neuronal cultures in the presence and absence of UNC0638 (250 nM) are shown. Vertical scale bar shows 0.025 (ΔF/F); horizontal bar shows 25 s. *N* = 300 ROIs for all cell lines across 2 imaging regions over 3 coverslips/line. The percentage of responsive cells and average calcium events/ROIs for control untreated hiPSCs and cells treated with UNC0638. **B** qRT-PCR analysis to examine changes in the expression of GluN1, GRIA1 and GRIN2A in EHMT1^−/+^-derived neurons relative to their expression in isogenic control neurons. The expression of GRIN1 was significantly elevated in EHMT1^−/+^ cells, while those of GRIA1 and GRIN2A were unchanged, *n* ≥ 3. Error bars represent SEM, all data points shown, *P < 0.05. **C** Equivalent stage neurons derived from KS-patient iPSC and control hiPSC were stained for neuronal marker MAP2 and the synaptic marker Synaptophysin positive puncta normalised to the dendritic area stained positive for MAP2. No significant difference in synaptic density, measured by the number of synaptophysin-stained puncta per unit area of MAP2 stained neurite, was observed. Representative images of Synaptophysin (SYN, green) positive puncta in MAP2 (red) positive control, KS1 and KS2 neurons. Scale bar = 50 µm. *n* ≥ 3 independent experiments. Data shown as Mean±SEM and analysed by student’s t-test or One-way ANOVA with post hoc comparisons using Dunnett’s multiple comparisons test comparing to control samples.

We next examined whether the abnormal developmental programme seen in *EHMT1*^+/−^ mutant cells altered neuronal function. To investigate neuronal activity, but minimise the impact of early neurodevelopmental deficits, differentiated wild-type cultures were switched BrainPhys medium [58] at Day 35 as they begin to form neurons to in the presence or absence of 250 nM UNC0638. Spontaneous calcium influx was measured two weeks later when the neurons typically begin to exhibit firing action potentials [59]. The number of spontaneously active cells and the frequency of calcium events per neuron was significantly higher in cells treated with UNC0638 compared to the untreated culture (Fig. 6A). This argues for a requirement of EHMT1 activity beyond Day 35.

Previously, Frega and co-workers (2019) described aberrant network activity of neurons derived from KS patient iPSCs that is driven by NMDA receptor (NMDAR) hyperactivity and is accompanied by elevated expression of the NMDAR subunit 1 (*GluN1*) gene [60]. We investigated changes in the expression of GluN1, the other NMDAR subunit *GluN2A* and an AMPAR subunit *GRIA1*. As in the previous report, we observed a significant upregulation in the expression of GluN1 mRNA in EHMT1^−/+^ iPSCs-derived neuron (Fig. 6B). Elevated GluN1 may lead to aberrant NMDA-mediated glutamate signalling. Expression of GRIA1 did not increase and in contrast to our observations in mESC, we observed little change for GluN2A. Finally consistent with previous results, we observed no change in the synaptic density of KS patient iPSC-derived neurons (Fig. 6C) [60].

## Discussion

Here, we report a regulatory pathway that connects the molecular lesion in EHMT1 activity to altered neuronal cell development and neuronal function in human iPSC-derived neurons. The key mechanistic components of the pathway are the transcriptional regulator NRSF/REST and its control via miRNA, with reduced EHMT1-mediated H3K9me2 resulting in elevated miRNA transcription. As a consequence, gene expression of both NRSF/REST-regulated and general neuronal specific markers is elevated with lower levels of EHMT1 activity but can be reduced to control levels by expression of a NRSF/REST mRNA that lacks the miRNA regulation sites. Although KS presents a strong genetically penetrant case for this pathway, our genetic association analysis suggests a broader association for ID and schizophrenia, with implications for therapeutic intervention.

NRSF/REST plays a key role in repression of neuronal gene expression to maintain stem cells in the undifferentiated state [28, 48], making it a major regulator of neurogenesis and neural differentiation [61]. Here, we can explain the in vitro cell phenotype seen in KS patient cells as due to reduced NRSF/REST protein. Our observations fit with the previous investigation of NRSF/REST hypofunction in neurodevelopment [28]. In the mouse brain, conditional NRSF/REST knockout mice show rapid neuronal differentiation of hippocampal neural stem cells and elevation in the expression of pro-neuronal genes, *NeuroD1, Tuj1, and DCX* [56]. In the study reported here, we show that reduced NRSF/REST expression in EHMT1^−/+^ cells is associated with elevated expression of the human pro-neural transcription factors MASH1 and NGN2.

The linkage between NRSF/REST and mental health is not well explored, but there are some mechanistic observations reported. In human neuronal culture, decreased nuclear NRSF/REST has been observed in neuronal cultures derived from sporadic Alzheimer’s Disease (AD) patient cells and again leads to accelerated neural differentiation and increased excitability, which can be reversed by exogenous NRSF/REST expression [62]. In the context of NDD, Down’s Syndrome cells have increased expression of DYRK1A which leads to reduced NRSF/REST and misregulation of neurodevelopmental genes [55]. Likewise, suppression of Chromodomain helicase DNA‐binding protein 2 (CHD2), associated with a range of NDD, including ASD and ID, was shown to inhibit the self-renewal of radial glial cells and increase the generation of neural progenitors and neurons and this phenotype was attributed to the reduced expression of the neuronal regulator NRSF/REST [63]. The exact mechanism leading to cell death of EHMT1^+/−^ hiPSC at these later stages of neurodifferentiation is unclear, but it is noteworthy that NRSF/REST-suppressed genes include cell death-inducing genes that may directly induce apoptosis [64]. We also note that reports in the mouse brain studies indicate that loss of *ehmt1* [65] or *rest* genes increase cell proliferation and adult neurogenesis, but the prolonged loss of NRSF/REST leads to a functional depletion of the adult neuronal stem cells and decreased granule neuron production [56]. Finally, analysis of post-mortem AD, where NRSF/REST is reduced compared to age-matched controls, show elevation of NRSF/REST targets, including genes encoding pro-apoptotic signalling components, associated with neurodegeneration [64].

Nonetheless, GWAS has not strongly associated NRSF/REST with psychiatric disorders. This may be because of a combination of network robustness that protects against minor fluctuations of its upstream regulatory pathway, and the severity caused by major changes in NRSF/REST expression, as reported with its association with dementias (AD, Huntington’s Disease and Parkinson’s Disease), ischemic shock and some NDD. Our cross-disorder analysis of the miRNA under EHMT1-regulation and their association with GWAS-significant miRNA genes suggests hitherto unexplored linkage with NSRF/REST not only for KS but across a broader range of ID and schizophrenia cases and may offer significant insights for alternative therapeutic strategy.

In summary, this study identifies a mechanism that couples EHMT1 activity to the neuronal regulator NRSF/REST through miRNA-dependent pathway, which leads to altered neurodevelopment. It suggests NRSF/REST as a key node within a miRNA-mediated gene regulatory network and offers a mechanism for the specific case of KS. Importantly it also reveals the presence of a more extensive pathway centred around NRSF/REST regulation of the neurodevelopmental gene regulation programme, which has broader significance for neurodevelopmental and psychiatric disorders, such as ID and schizophrenia.

## Supplementary information

Supplemental Material
