# Supplementary material for: NRSF/REST lies at the intersection between epigenetic regulation, miRNA-mediated gene control and neurodevelopmental pathways associated with Intellectual disability (ID) and Schizophrenia
Source: Transl Psychiatry. 2022 Oct 10;12:438. doi: 10.1038/s41398-022-02199-z (PMC9551101; doi:10.1038/s41398-022-02199-z)
Supplement: Supplementary file 1 — Supplemental Material [file 41398_2022_2199_MOESM1_ESM.docx]

**NRSF/REST lies at the intersection between epigenetic regulation, miRNA-mediated gene control and neurodevelopmental pathways associated with Intellectual disability (ID) and Schizophrenia**

Mouhamed Alsaqati, Brittany A Davis, Lora Jones, Aishah Westwood, Olena Petter, Anthony R Isles, David Linden, Marianne Van den Bree, Michael Owen, Jeremy Hall, Adrian J Harwood,

**Supplemental Data**

**Table S1**

**Table S1: mRNA analysis of selected NRSF/REST- regulated genes of mouse ESC (mESC) in early stages of in vitro differentiation (Day 8).**

| **Gene name (mouse)** | **Human orthologue** | **Fold change** | **p-value** | |
| --- | --- | --- | --- | --- |
| Bdnf | BDNF | 1.22 | 0.408 | ns |
| Ctnnd2 | Catenin delta 2 | 5.04 | 0.012 | * |
| Grin2a | GluN2A | 6.872 | 0.043 | * |
| L1cam | NCAM L1 | 15.21 | 0.014 | * |
| Nefh | Neurofilament H | 1.315 | 0.22 | ns |
| Notch4 | NOTCH4 | 5.605 | 0.034 | * |
| Nrxn3 | Neurexin 3 | 3.24 | 0.014 | * |
| Ntrk3 | NTRK3 | 3.244 | 0.017 | * |
| Smad2 | SMAD2 | 1.034 | 0.113 | ns |
| Snap25 | SNAP25 | 122.9 | 0.002 | ** |
| Syn1 | Synapsin 1 | 5.412 | 0.004 | ** |
| Syp | Synaptophysin | 13.01 | 0.003 | ** |
| Stmn2 | Stathmin 2 | 98.9 | 0.001 | *** |
| Rest | NRSF/REST | 0.161 | 0.001 | *** |

Mean of fold change over control untreated hiPSCs,

n ≥ 3 independent experiments.

p-values: *** <0.001; ** <0.01; * <0.05 *; ns: no significance.

For full analysis see https://orca.cardiff.ac.uk/id/eprint/73068/

**Table S2**

Potential off-target sites for EHMT1SygRNA

| **Target** | **Potential off-target sites** | **Strand** | **Chr.No.** | **Start coordinate** | **Position of mismatches** | **Mismatch count** |
| --- | --- | --- | --- | --- | --- | --- |
| 1 | AGCCGGGACACCAGTCCCCGGG | + | Chr7 | 1733249 | 0:A>G,1:G>A,5:G>T | 3 |
| 2 | GTTCCTGACACCAGTCCCCGGG | + | Chr11 | 128869635 | 1:T>A,2:T>C,4:C>G | 3 |
| 3 | GGCCGTGACACCAGTGCCTGGG | + | Chr14 | 97456999 | 1:G>A,15:G>C,18:T>C | 3 |
| 4 | GACCGTGACACCAGTCTCTGGG | + | Chr9 | 134427650 | 3:G>C,12:T>A,14:C>T | 3 |

PCR primers for amplifying predicted off-target sites for EHMT1SygRNA

| **Oligo name** | **Sequence (5'-3')** | **Product size (bp)** |
| --- | --- | --- |
| Target_ 1 F  Target_ 1 R | GCTGAACAAGCTTCGGGGAA  GGACAGGGAAGGGATTTGCT | 573 |
| Target_ 2 F  Target_ 2 R | CTGTCCCCTCCTCCACTCAA  GCCTTTTCCGTGAAATTTTGCAT | 213 |
| Target_ 3 F  Target_ 3 R | ACTGCACTCATAGGTCCCCT  AGGTGCAATGTGCCTTCAGA | 354 |
| Target_ 4 F  Target_ 4 R | GTGACGGTATTTCCAGCCCT  CAGCAAGAAGTTGGTGGGGA | 345 |

**Table S3**

Primers for mature miRNA quantitation.

| **Oligo name** | **mature miRNA sequence** |
| --- | --- |
| hsa-miR-181a-1 | AACATTCAACGCTGTCGGTGAGT |
| hsa-miR-142 | TGTAGTGTTTCCTACTTTATGGA |
| hsa-miR-153-1 | TTGCATAGTCACAAAAGTGATC |
| hsa-miR-769 | CTGGGATCTCCGGGGTCTTGGTT |
| hsa-miR-26a-2 | CCTGTTCTTGATTACTTGTTTC |
| hsa-miR-340 | TCCGTCTCAGTTACTTTATAGC |
| hsa-miR-378a | ACTGGACTTGGAGTCAGAAGG |
| hsa-miR-26b | CCTGTTCTCCATTACTTGGCTC |
| hsa-miR-548f-1 | AAAAACTGTAATTACTTTT |
| hsa-miR-3607 | ACTGTAAACGCTTTCTGATG |
| hsa-miR-125b2 | ACGGGTTAGGCTCTTGGGAGCT |

Primers for gene quantitation.

| **Gene name** | **Forward (5'-3') &Reverse (5'-3')** |
| --- | --- |
| Ngn2 | TTTGCAATGGCTGGCATCT & CACAGCCTGCAGACAGCAAT |
| Mash1 | AACGAGCGCGAGCGCAACCG & TTGGAGTAGTTGGGGGAGATG |
| Map-2 | CCACCTGAGATTAAGGATCA & GGCTTACTTTGCTTCTCTGA |
| Nestin | TCCAGAAACTCAAGCACCA & AAATTCTCCAGGTTCCATGC |
| NCAM | ACATCACCTGCTACTTCCTG & CTTGGACTCATCTTTCGAGAAGG |
| NRXN3 | GCTGAGAACAACCCCAATA & ATGCTGGCTGTAGAGCGATT |
| ACTA1 | AGGTCATCACCATCGGCAACGA & GCTGTTGTAGGTGGTCTCGTGA |
| Calbindin | TGGCATCGGAAGAGCAGCAG & TGACGGAAGTGGTTACCTGGAAG |
| hGAPDH | AGGCTGGGGCTCATTTG & CAGTTGGTGGTGCAGGAG |
| Colrf43 | GGCATCCCCGTTCCTTAATGG & TACACCCTTGAAAGGCGTACT |
| mGAPDH | GAACATCATCCCTGCATCCA & CCAGTGAGCTTCCCGTTCA |
| GluN1 | CGCCGCTAACCATAAACAAC & GGGGAATCTCCTTCTTGACC |
| GRIA1 | GCAGCAGTGGAAGAATAGTGATG & ATCACCTTCACCCCATCGTA |
| GRIN2A | AGCTGCTACGGGCAGATG & CCTGGTAGCCTTCCTCAGTG |
| Caspase-3 | TCGGTCTGGTACAGATGTCG & CATACAAGAAGTCGGCCTCC |

**Table S4**

primers for CHIP-PCR

| **Oligo name** | **Forward (5'-3') &Reverse (5'-3')** |
| --- | --- |
| miR-142  P1  P2  P3 | GAACACACCAGGGCCTCAAT & TGTTGGGATAGCCTTGGGC  GGGTCCCGGAGCCATTTTAC & GAAGGGAACCCCACTTCGG  CTAAGGAGGCCTGGGTCGAT & GCCGTGAGAAACCTGCTCTT |
| miR-153  P1  P2  P3 | CTGTGCCACCTCGAGGCTAT & TGAACTTAAGAAGCTCACTCCCC  GTAAGAGCCCTGTCGGAGC & CGGGTTTATCGTTCTGGACC  GGGTGATGTTACGAGGGTCA & GCCTGGGTATAGGGCAGAATC |
| miR-26a  P1  P2  P3 | CACACTCGTCCTTACACCCC & AAGTGCGAGTTCTCTGTGGG  CGAATCCCGAAAGGGGTGTGT & TAAATGGGAGTAAGTCGGTGGGT  GGGATTGCCGAAAGACCAAG & GCTGGGTTAGGTTAGGACCC |
| REST promotor  P1  P2  P3 | GGGTGGCAGTTAGGGGTTAG & AAGGTGCCATTCTCAAACGAA  ACTTCGTTTGAGAATGGCACC&TCGTGCAACTCAACTCGACA ATACACTTGGGTGAAGCGGG & GGTGCCATTCTCAAACGAAGT |

**Table S5.**

Hypergeometric p-values for overlap between miRNA gene targets and known disease related genes identified by GWAS.

| **miRNA** | **Fold Change** | **ID** | **SCZ** | **ASD** |
| --- | --- | --- | --- | --- |
| hsa-mir-653 | 4.00 | 3.25E-07 | 4.20E-02 | 0.026 |
| hsa-mir-181a-1 | 3.69 | 8.42E-05 | 2.56E-06 | 6.51E-05 |
| hsa-mir-5688 | 3.43 | 1.76E-12 | 9.41E-09 | 1.57E-06 |
| hsa-mir-142 | 3.37 | 5.78E-08 | 2.44E-05 | 0.027 |
| hsa-mir-139 | 3.16 | 8.57E-05 | 9.49E-04 | 0.081 |
| hsa-mir-627 | 3.12 | 5.48E-05 | 0.168 | 0.105 |
| hsa-mir-125b-2 | 3.10 | 0.01 | 0.062 | 0.021 |
| hsa-mir-193a | 3.09 | 0.008 | 2.00E-03 | 0.051 |
| hsa-mir-205 | 3.06 | 3.07E-06 | 4.13E-06 | 0.098 |
| hsa-mir-2276 | 3.05 | 0.011 | 3.41E-05 | 0.398 |
| hsa-mir-188 | 2.99 | 1.13E-05 | 0.027 | 1.00E-03 |
| hsa-mir-1287 | 2.98 | 0.103 | 0.053 | 0.178 |
| hsa-mir-548f-1 | 2.97 | 4.04E-18 | 3.27E-05 | 1.90E-08 |
| hsa-mir-548ao | 2.95 | 0.053 | 0.0261 | 0.268 |
| hsa-mir-769 | 2.91 | 0.004 | 0.002 | 0.46 |
| hsa-mir-331 | 2.86 | 0.221 | 0.493 | 0.368 |
| hsa-mir-3117 | 2.86 | 0.013 | 0.001 | 0.149 |
| hsa-mir-30b | 2.84 | 5.20E-07 | 3.36E-07 | 4.54E-04 |
| hsa-mir-29c | 2.84 | 1.46E-07 | 0.028 | 0.087 |
| hsa-mir-545 | 2.82 | 5.62E-10 | 5.71E-04 | 0.117 |
| hsa-mir-489 | 2.82 | 0.008 | 0.053 | 0.404 |
| hsa-mir-153-1 | 2.79 | 1.23E-11 | 6.03E-07 | 7.30E-04 |
| hsa-mir-4664 | 2.79 | 0.111 | 0.084 | 0.126 |
| hsa-mir-183 | 2.79 | 6.73E-05 | 6.75E-04 | 0.003 |
| hsa-mir-26b | 2.77 | 1.90E-07 | 0.002 | 2.85E-06 |
| hsa-mir-378a | 2.76 | 6.00E-03 | 0.01 | 0.11 |
| hsa-mir-374a | 2.76 | 5.18E-05 | 5.40E-02 | 0.026 |
| hsa-mir-107 | 2.75 | 0.005 | 6.45E-04 | 0.005 |
| hsa-mir-26a-2 | 2.73 | 2.75E-08 | 1.61E-05 | 1.78E-07 |
| hsa-mir-4424 | 2.72 | 0.134 | 1.75E-04 | 0.498 |
| hsa-mir-3607 | 2.71 | 0.003 | 0.007 | 0.151 |
| hsa-mir-590 | 2.71 | 1.10E-12 | 1.09E-04 | 0.011 |
| hsa-mir-497 | 2.71 | 4.18E-09 | 8.00E-03 | 0.044 |
| hsa-let-7e | 2.70 | 0.011 | 0.006 | 0.205 |
| hsa-mir-592 | 2.69 | 0.019 | 3.00E-03 | 0.38 |
| hsa-mir-1228 | 2.67 | 2.29E-07 | 0.021 | 0.105 |
| hsa-mir-340 | 2.66 | 7.19E-06 | 2.64E-06 | 0.075 |
| hsa-mir-542 | 2.64 | 0.003 | 0.42 | 0.049 |
| hsa-mir-577 | 2.64 | 1.79E-05 | 2.21E-04 | 0.215 |
| hsa-mir-1468 | 2.64 | 9.38E-09 | 1.39E-04 | 0.058 |
| hsa-mir-598 | 2.63 | 0.438 | 0.215 | 0.11 |
| hsa-mir-27b | 2.62 | 8.96E-05 | 1.90E-02 | 0.05 |
| hsa-mir-4677 | 2.61 | 0.006 | 0.005 | 0.06 |
| hsa-mir-561 | 2.60 | 1.77E-08 | 8.41E-06 | 1.52E-06 |
| hsa-mir-3943 | 2.58 | 0.28 | 0.129 | 0.264 |
| hsa-mir-3651 | 2.57 | 0.473 | 0.385 | 0.15 |
| hsa-mir-424 | 2.55 | 8.18E-10 | 3.00E-03 | 0.007 |
| hsa-mir-1229 | 2.54 | 0.074 | 0.482 | 0.453 |
| hsa-mir-335 | 2.53 | 8.76E-09 | 1.05E-06 | 0.008 |
| hsa-mir-4661 | 2.53 | 7.23E-05 | 1.80E-02 | 0.071 |
| hsa-mir-574 | 2.53 | 0.002 | 6.41E-06 | 0.02 |
| hsa-mir-1306 | 2.52 | 1.61E-05 | 0.106 | 0.371 |
| hsa-mir-5010 | 2.52 | 4.53E-12 | 1.27E-06 | 0.003 |
| hsa-mir-1303 | 2.51 | 0.003 | 0.11 | 0.317 |
| hsa-mir-33b | 2.51 | 4.74E-07 | 4.91E-05 | 0.003 |
| hsa-mir-140 | 2.51 | 2.21E-06 | 0.05 | 0.453 |

miRNAs predicted to target the REST gene are highlighted in grey

**Table S6.**

miRNAs with significant overlap between miRNA target genes and disease related genes identified by GWAS

| **miRNA** | **Fold Change** | **ID** | **SCZ** | **ASD** |
| --- | --- | --- | --- | --- |
| hsa-mir-653 | 4.00 | YES |  |  |
| hsa-mir-181a-1 | 3.69 | YES | YES | YES |
| hsa-mir-5688 | 3.43 | YES | YES | YES |
| hsa-mir-142 | 3.37 | YES | YES |  |
| hsa-mir-139 | 3.16 | YES | YES |  |
| hsa-mir-627 | 3.12 | YES |  |  |
| hsa-mir-125b-2 | 3.10 | YES |  |  |
| hsa-mir-193a | 3.09 | YES | YES |  |
| hsa-mir-205 | 3.06 | YES | YES |  |
| hsa-mir-2276 | 3.05 |  | YES |  |
| hsa-mir-188 | 2.99 | YES |  | YES |
| hsa-mir-1287 | 2.98 |  |  |  |
| hsa-mir-548f-1 | 2.97 | YES | YES | YES |
| hsa-mir-548ao | 2.95 |  |  |  |
| hsa-mir-769 | 2.91 | YES | YES |  |
| hsa-mir-331 | 2.86 |  |  |  |
| hsa-mir-3117 | 2.86 |  | YES |  |
| hsa-mir-30b | 2.84 | YES | YES | YES |
| hsa-mir-29c | 2.84 | YES |  |  |
| hsa-mir-545 | 2.82 | YES | YES |  |
| hsa-mir-489 | 2.82 | YES |  |  |
| hsa-mir-153-1 | 2.79 | YES | YES | YES |
| hsa-mir-4664 | 2.79 |  |  |  |
| hsa-mir-183 | 2.79 | YES | YES | YES |
| hsa-mir-26b | 2.77 | YES | YES | YES |
| hsa-mir-378a | 2.76 | YES | YES |  |
| hsa-mir-374a | 2.76 | YES |  |  |
| hsa-mir-107 | 2.75 | YES | YES | YES |
| hsa-mir-26a-2 | 2.73 | YES | YES | YES |
| hsa-mir-4424 | 2.72 |  | YES |  |
| hsa-mir-3607 | 2.71 | YES | YES |  |
| hsa-mir-590 | 2.71 | YES | YES |  |
| hsa-mir-497 | 2.71 | YES | YES |  |
| hsa-let-7e | 2.70 |  | YES |  |
| hsa-mir-592 | 2.69 |  | YES |  |
| hsa-mir-1228 | 2.67 | YES |  |  |
| hsa-mir-340 | 2.66 | YES | YES |  |
| hsa-mir-542 | 2.64 | YES |  |  |
| hsa-mir-577 | 2.64 | YES | YES |  |
| hsa-mir-1468 | 2.64 | YES | YES |  |
| hsa-mir-598 | 2.63 |  |  |  |
| hsa-mir-27b | 2.62 | YES |  |  |
| hsa-mir-4677 | 2.61 | YES | YES |  |
| hsa-mir-561 | 2.60 | YES | YES | YES |
| hsa-mir-3943 | 2.58 |  |  |  |
| hsa-mir-3651 | 2.57 |  |  |  |
| hsa-mir-424 | 2.55 | YES | YES | YES |
| hsa-mir-1229 | 2.54 |  |  |  |
| hsa-mir-335 | 2.53 | YES | YES | YES |
| hsa-mir-4661 | 2.53 | YES |  |  |
| hsa-mir-574 | 2.53 | YES | YES |  |
| hsa-mir-1306 | 2.52 | YES |  |  |
| hsa-mir-5010 | 2.52 | YES | YES | YES |
| hsa-mir-1303 | 2.51 | YES |  |  |
| hsa-mir-33b | 2.51 | YES | YES | YES |
| hsa-mir-140 | 2.51 | YES |  |  |
|  | Total | **43** | **34** | **15** |

“YES” indicates a significant overlap (Hypergeometric probability; p<0.01) between disease associated genes and miRNA gene targets

miRNAs predicted to target the REST gene are highlighted in grey

**Supplemental Figures**

**Figure S1: Characterisation of KS patient iPSC.**

**
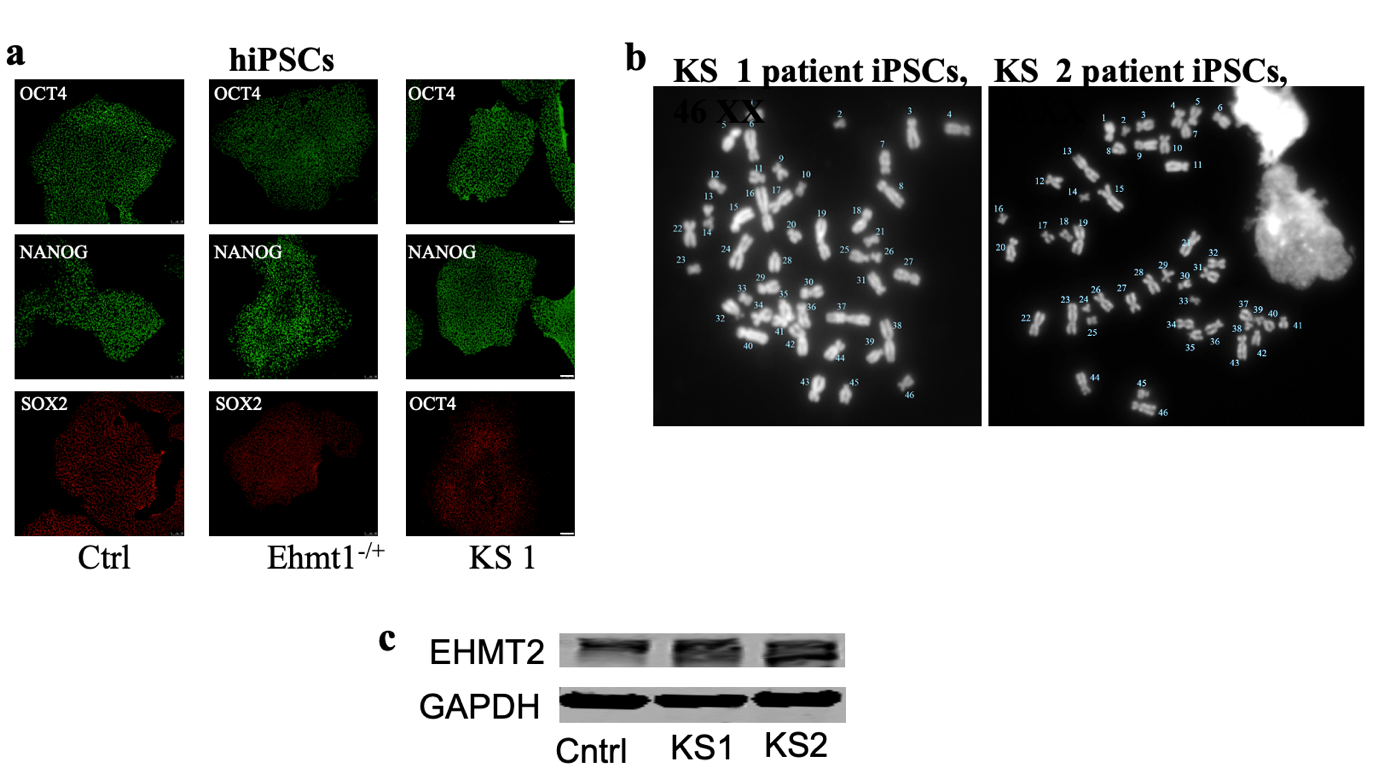
**

**a**, Characterisation of hiPSC line. Morphology of control hiPSCs, EHMT1^-/+^-iPSCs and KS1 patient iPSCs expressing stem cell markers OCT4 (green), nanog (green) and Sox2 (red). Images were acquired using Leica DMI6000b fluorescent microscope. Scale bar, 100µM. **b**, Normal karyotype generated from human female KS1 and KS2 patient iPSCs showed no chromosomal aberration in all analysed metaphases (46, XX) (×100). **c,** No expression change of EHMT2 was observed

**Figure S2: Generation of isogenic EHMT1^+/-^ hiPSC line**

**
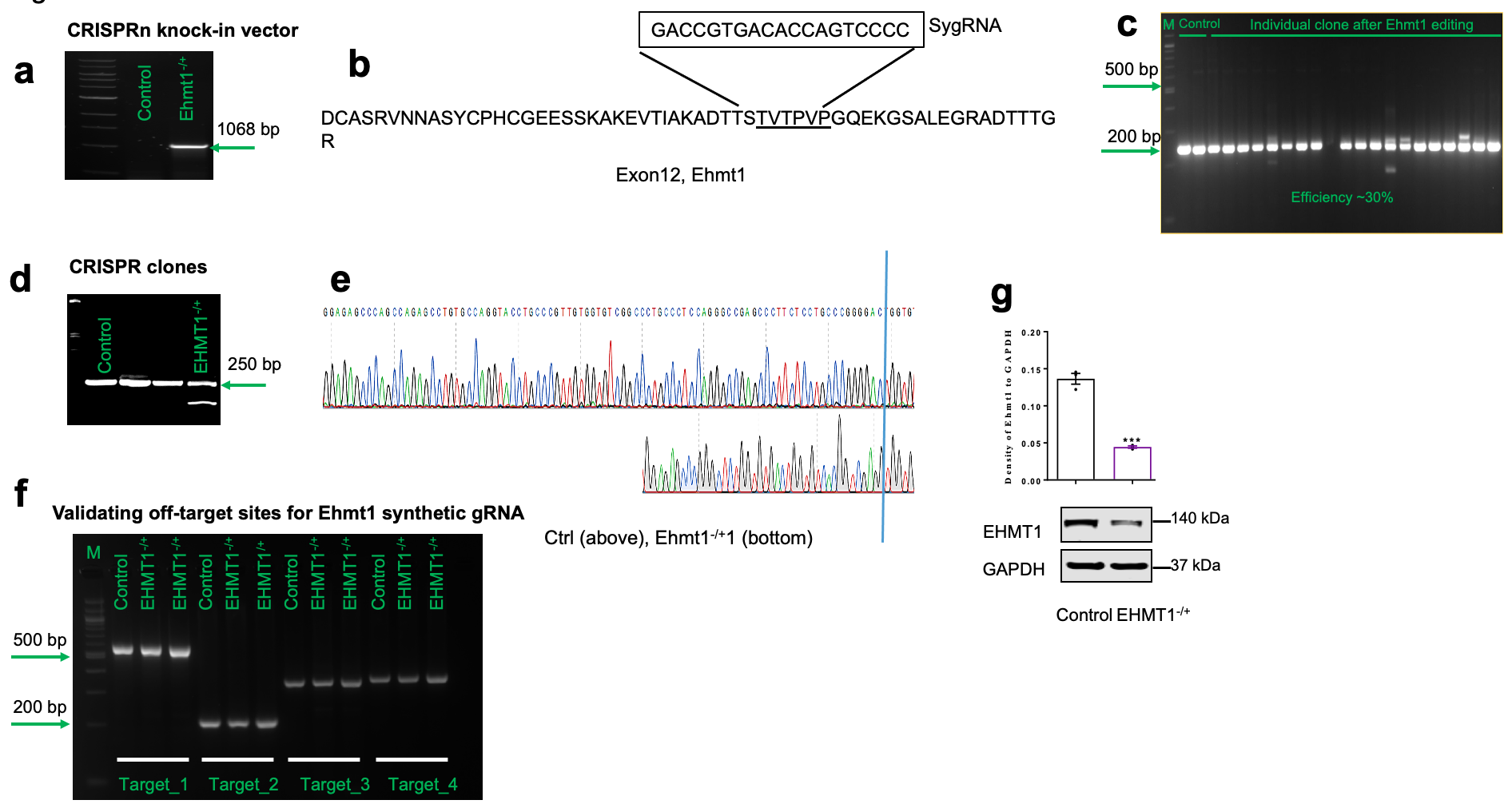
**

**a**, Junction PCR confirmed on-target integration into the AAVS1 locus of the CRISPRn clones. **b**, Design of CRISPR SygRNA sequence targeting the exon12 of EHMT1 gene. **c,** A 250-bp PCR amplicon encompassing the CRISPR target in exon 12 was PCR amplified from genomic DNA using primers 5`-AGCAGCATCTCTCACCGTTT-3` and 5`-CTTTTTCAGGTGGACGACTGG-3`, and PCR products were size-separated by electrophoresis on a 4% agarose gel. Several clones were potentially edited with ~30% efficiency, and (**d.**) subcloned to obtain a clone with 56-bp deletion in exon12. M: DNA marker. **e**, DNA-sequencing analysis of EHMT1^-/+^ iPSC clone. Knockout alleles were identified by amplifying the PCR product containing the edit by PCR and Sanger sequencing it using specific primers. Blue bar indicates the site that EHMT1 sequence difference between the control and the EHMT1^-/+^ iPSC clone appears. **f**, Off-target sites for the CRISPR/Cas9 EHMT1 SygRNA were predicted using an online tool. PCR primers were designed to amplify fragments for each off-target site, and the PCR products were run on a gel to detect whether any predicted off-target shows any indel. The gel picture represents the PCR products of the four predicted targets: none of the predicted off-target PCR products showed any extra band below or above, indicating that there is no detectable off-target cleavage occurred for the EHMT1 SygRNA. M: DNA marker. For each target, the bands of EHMT1^-/+^ cells were compared to those of the control cells. (**g**) Western blot analysis of EHMT1 protein expression was performed in EHMT1^-/+^ iPSC. Expression of EHMT1 was decreased by ~50% in EHMT1^-/+^ iPSC clone in relation to the isogenic control. Western blot analysis was performed by normalization to GAPDH, n ≥ 3. Error bars represent SEM, ****P* < 0.001.

**Figure S3: Schematic shows the positions of REST promoter and exons, and transcription start sites (TSSs) of miR-142, miR-153 and miR-26a.**


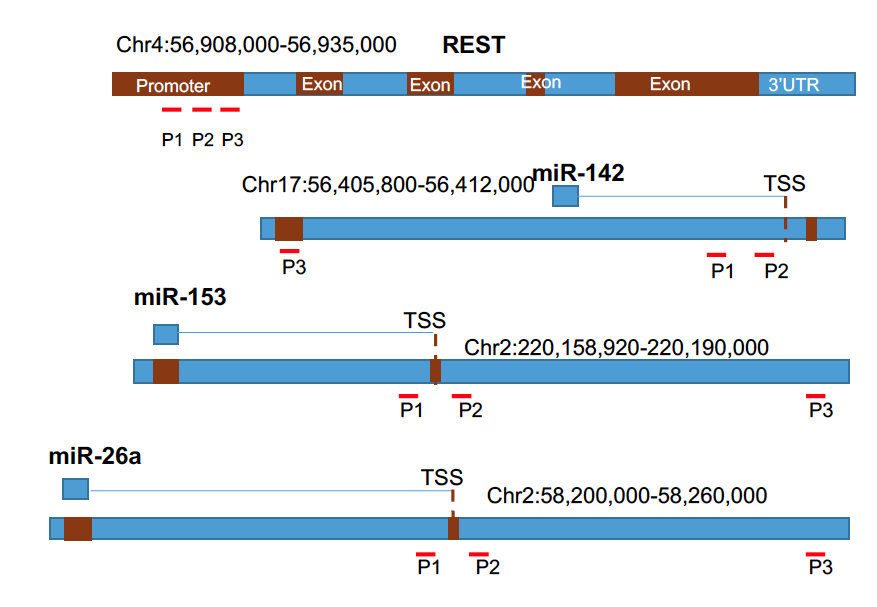


The positions of primers used in ChIP-qRT-PCR analysis are labelled in red. miRNAs positions were predicted from Ensembl. The TSSs of the miRNAs were obtained from [http://mirstart.mbc.nctu.edu.tw/browse.php](about:blank).

**Figure S4: miRNA-seq analysis**

c

miRNAs-Seq

Log2 Fold Change

**-log10 (Pvalue)**


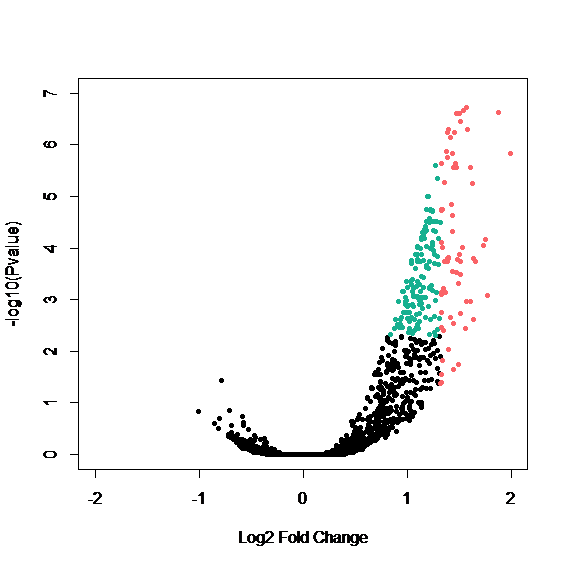


**
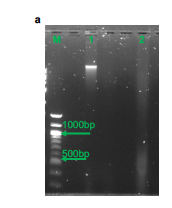

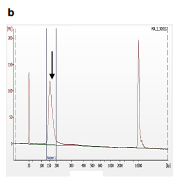
**


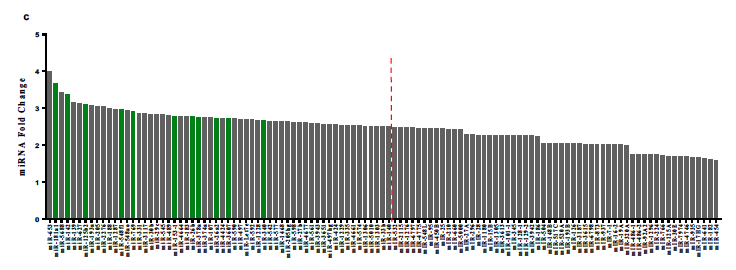


**a**, Chip samples were sheared for six rounds of sonication cycles of 30 sec ON/30 sec OFF with the Bioruptor® PLUS combined with the Minichiller® Water cooler (Cat. No. B02010003) at HIGH power setting. The sheared chromatin was de-crosslinked prior to agarose gel electrophoresis. 20µl of Sheared DNA (equivalent to 1µg DNA) were analysed on a 1% agarose gel. M: DNA marker, lane 1, non-sonicated DNA and lane 2, sonicated DNA for 30 min. Using the current setting, optimal chromatin fragments ranging from 200 to 600 bp are abundant in the ChIP sample. **b**, Visualization of miRNA sequencing libraries. Bioanalyser profile of miRNA sequencing library on high-sensitivity DNA Chip. The library (labelled with a black arrow) is ~150 bp in size and shows a clear, defined peak. c, A volcano plot of the differentially expressed miRNAs in hiPSCs treated with UNC0638 as compared to the control iPSCs. The data was analysed using DESeq2 algorithm and thresholds (cutoff) of ≥ 2.5-fold change in expression and adj.pval of < 0.05 were applied. While we did not detect any significantly downregulated miRNAs, 37 miRNAs were significantly upregulated above the threshold. Red dots represent significantly differentially expressed miRNAs following applying these criteria. Green points represent the miRNAs which were significantly increased but below the 2.5-fold change cutoff. **d**, Examples of differentially expressed miRNAs in the presence of UNC0638 in comparison to the untreated iPSCs, as assessed with the DESeq2 algorithm and Benjamini-Hochberg correction for multiple testing, adj.pval < 0.05). Red broken line indicates the cuttoff. Green bars represent REST-regulated miRNAs.

**Figure S5: Generation and characterization of RESTΔUTR-inducible hiPSC lines**.


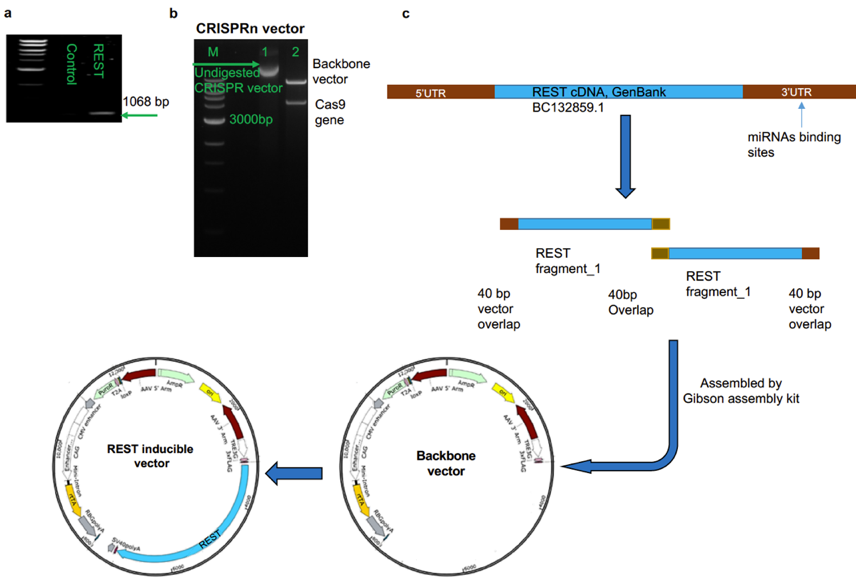


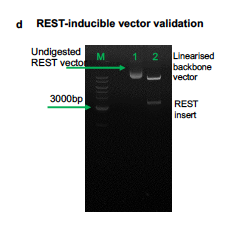

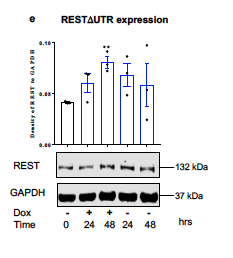


**a**, Junction PCR confirmed on-target integration into the AAVS1 locus of REST-inducible clones. **b**, Agarose gel electrophoresis (1%) of CRISPRn plasmid digested with AfIII and AgeI restriction enzymes. M: DNA marker, lane 1, undigested CRISPRn plasmid and lane 2, the CRSIPRn plasmid digested with AfIII and AgeI, as well as the Cas9 restriction fragment. **c**, Schematic representation of the workload carried out to generate REST-inducible vector. REST cDNA sequence lacking the 3’UTR was obtained from NCBI, GenBank BC132859.1. The sequence of ~3000bp was synthetised as two fragments (~1500-bp each) of a double-strand DNA using IDT standard procedure. REST two fragments as well as the linearised backbone vector with 40-bp overlaps either side were assembled in an isothermal Gibson assembly reaction following the manufacturer’s instructions. REST-inducible vector was generated. **d**, Successful assembly of REST fragments into the backbone vector was validated using restriction digestion of REST-inducible vector. Agarose gel electrophoresis (1%) of REST-inducible vector digested with AfIII and AgeI restriction enzymes was carried out, and the results confirmed the successful cloning. M: DNA marker, lane 1, undigested REST-inducible vector and lane 2, the linearized digested REST-inducible vector, as well as REST restriction fragment. **e**, Western blot analysis of REST protein after 2μg/mL doxycycline (Dox) treatment. Quantification of Western blot analysis was performed by normalization to GAPDH. The expression of REST protein was enhanced following DOX treatment for 48h and it started to decrease after removing Dox. N ≥ 3 independent experiments, error bars represent SEM. **P* < 0.05, ***P* < 0.01.

**Figure S6:**

Histogram of hypergeometric p-values of overlap between miRNA target genes and disease related genes identified by GWAS

**
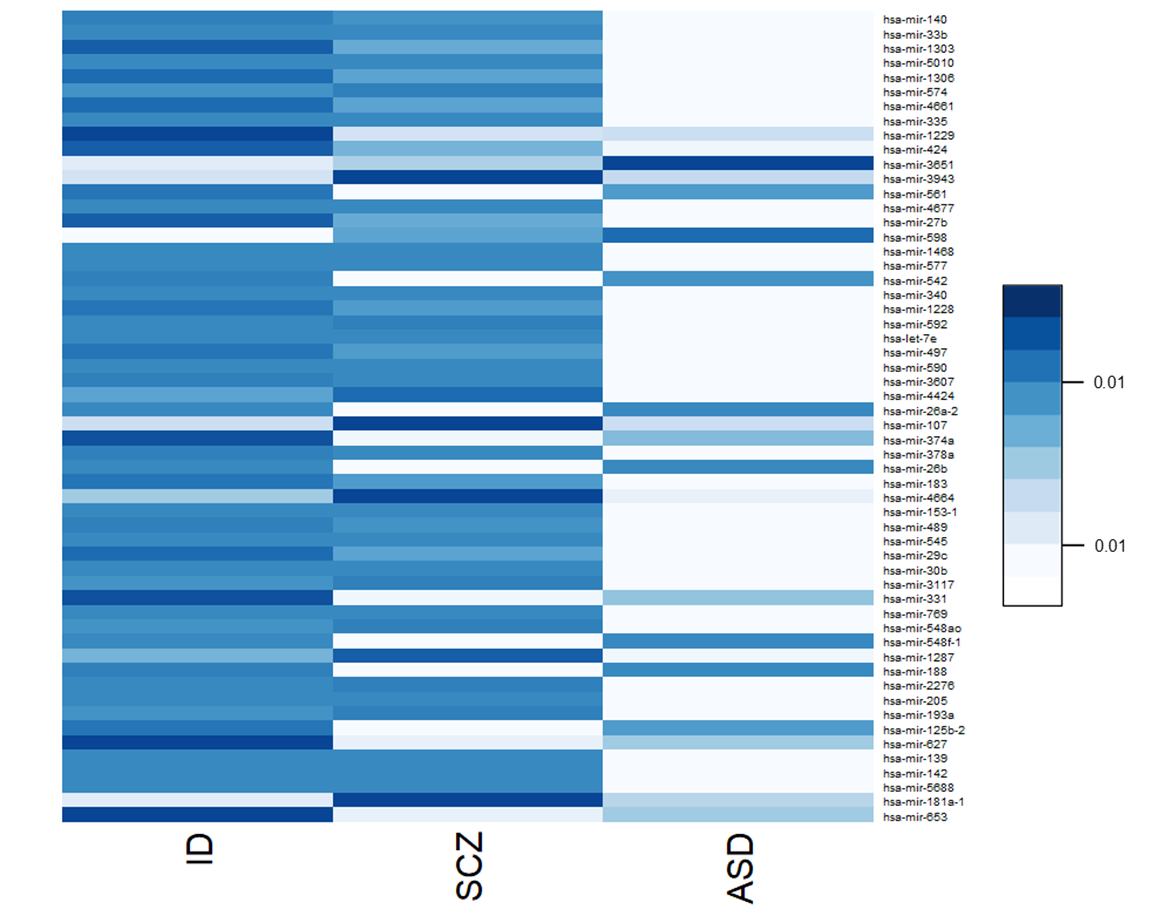
**

**Figure S7: Venn diagrams of overlap between miRNA target genes and disease related genes identified by GWAS**

**
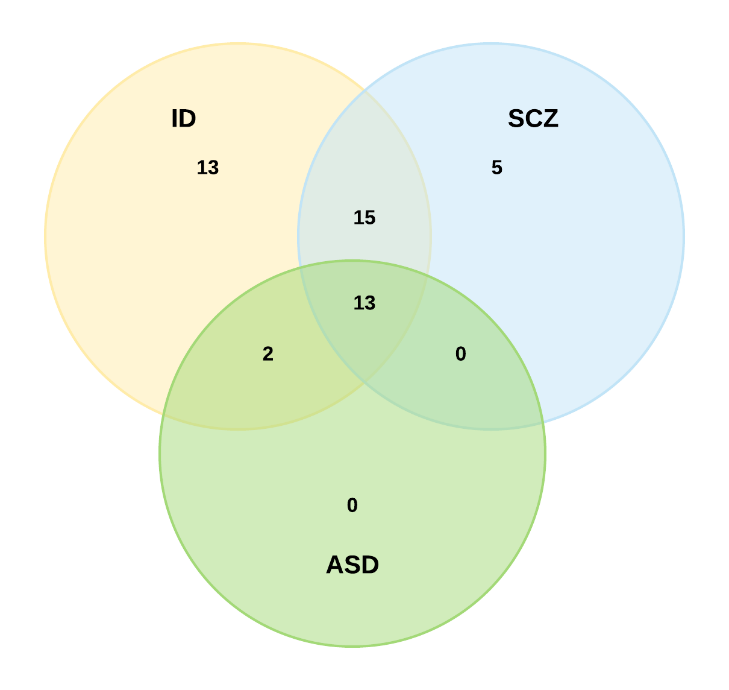

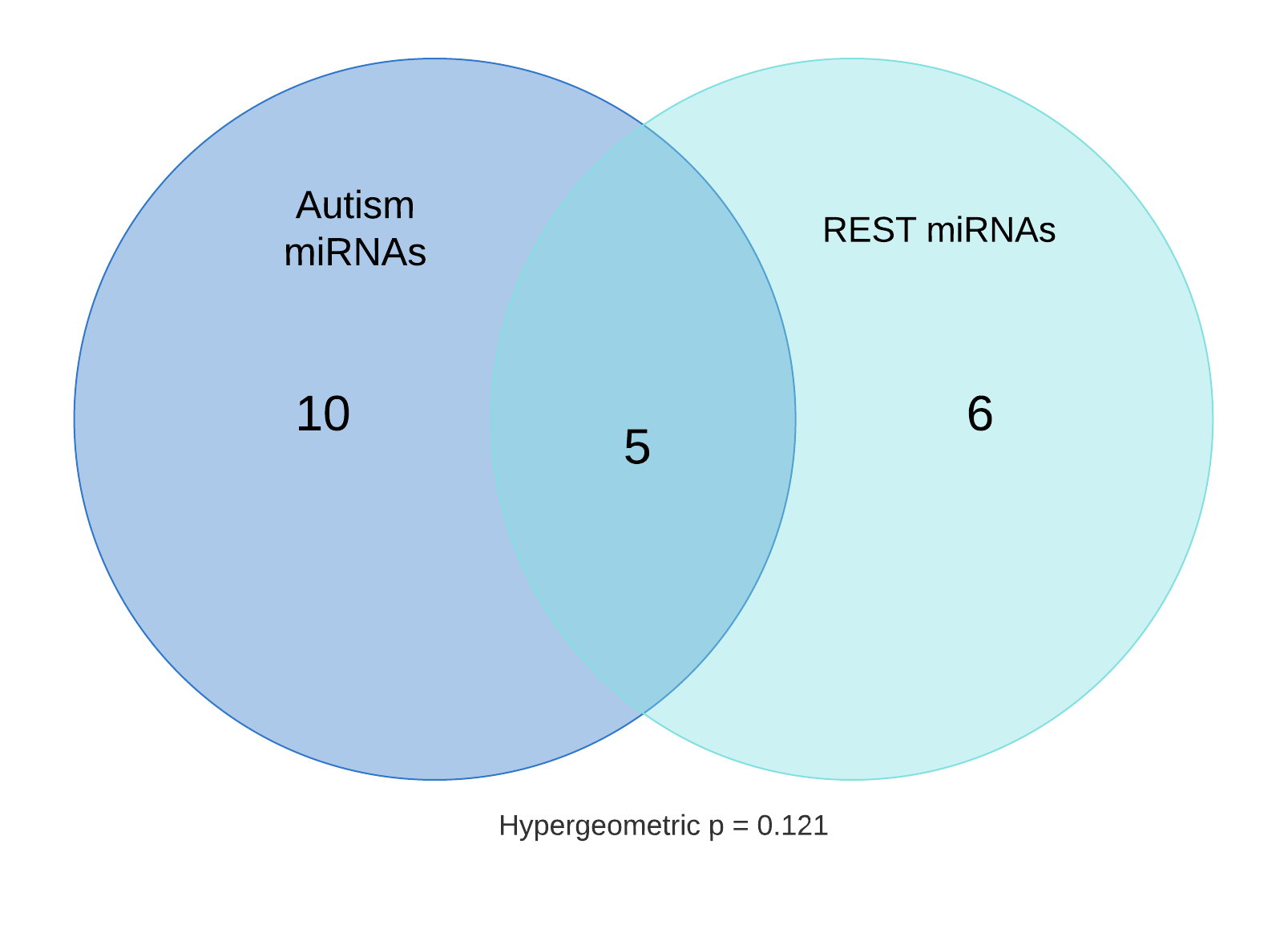
**

**A**

**B**

**a,** Venn diagram indicating overlap between miRNAs targeted to target the REST gene and miRNAs predicted to target a significant of Autism related genes, identified by GWAS. Hypergeometric analysis indicated the overlap (5 miRNAs) was not significant (p=0.121).

**b,** Venn diagram indicating overlap of miRNAs significantly targeting key genes associated with Intellectual Disability (ID), Schizophrenia (SCZ) and Autism (ASD).

**Figure S8: Late differentiation analysis of MAP2, Nestin and NCAM**

**
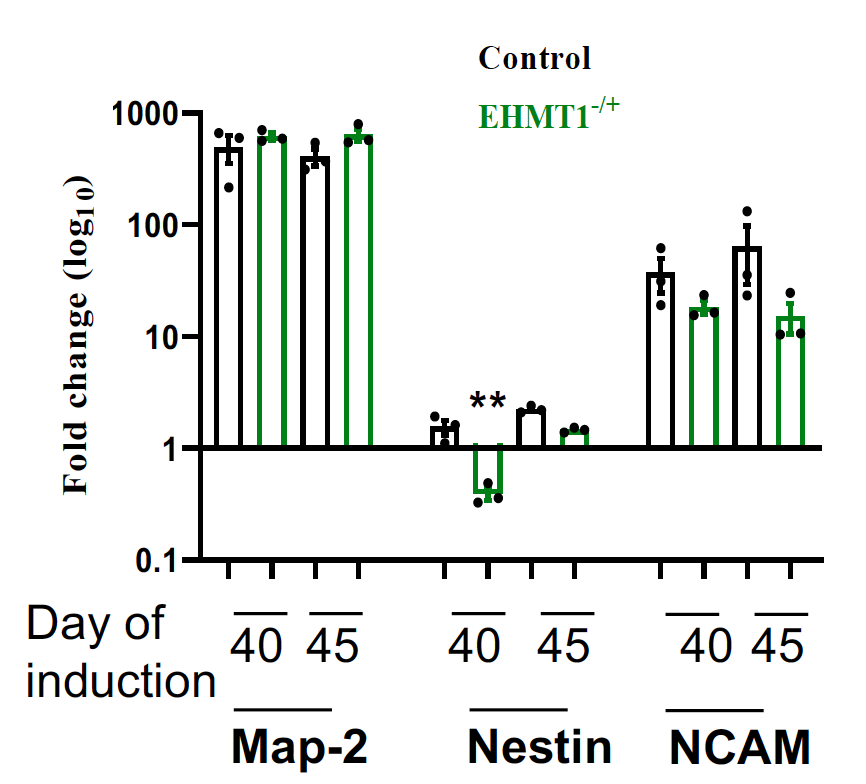
**

Time-course qRT-PCR analysis at days 40 and 45 of differentiation to examine changes in the expression of MAP2, Nestin and NCAM in hiPSCs-derived neurons in relation to the control. The expression of MAP2, Nestin or NCAM was slightly reduced in EHMT1^+/-^ cells.
